# A Genetic Map Between *Gossypium hirsutum* and the Brazilian Endemic *G. mustelinum* and Its Application to QTL Mapping

**DOI:** 10.1534/g3.116.029116

**Published:** 2016-03-31

**Authors:** Baohua Wang, Limei Liu, Dong Zhang, Zhimin Zhuang, Hui Guo, Xin Qiao, Lijuan Wei, Junkang Rong, O. Lloyd May, Andrew H. Paterson, Peng W. Chee

**Affiliations:** * Plant Genome Mapping Laboratory, University of Georgia, Athens, Georgia 30602; † School of Life Sciences, Nantong University, Nantong, Jiangsu 226019, China; ‡ Department of Crop and Soil Sciences, University of Georgia, Tifton, Georgia 31793; § School of Agriculture and Food Science, Zhejiang A&F University, Lin’an, Hangzhou, Zhejiang 311300, China

**Keywords:** map comparison, chromosome structural changes, phylogenetic context, colinearity

## Abstract

Among the seven tetraploid cotton species, little is known about transmission genetics and genome organization in *Gossypium mustelinum*, the species most distant from the source of most cultivated cotton, *G. hirsutum*. In this research, an F_2_ population was developed from an interspecific cross between *G. hirsutum* and *G. mustelinum* (HM). A genetic linkage map was constructed mainly using simple sequence repeat (SSRs) and restriction fragment length polymorphism (RFLP) DNA markers. The arrangements of most genetic loci along the HM chromosomes were identical to those of other tetraploid cotton species. However, both major and minor structural rearrangements were also observed, for which we propose a parsimony-based model for structural divergence of tetraploid cottons from common ancestors. Sequences of mapped markers were used for alignment with the 26 scaffolds of the *G. hirsutum* draft genome, and showed high consistency. Quantitative trait locus (QTL) mapping of fiber elongation in advanced backcross populations derived from the same parents demonstrated the value of the HM map. The HM map will serve as a valuable resource for QTL mapping and introgression of *G. mustelinum* alleles into *G. hirsutum*, and help clarify evolutionary relationships between the tetraploid cotton genomes.

The cotton genus *Gossypium* L. comprises more than 50 species, including eight diploid groups and seven tetraploid species (Fryxell 1992; Wendel and Cronn 2003; Wendel and Grover 2015). Two tetraploid species—*G. hirsutum* L. (AD1) and *G. barbadense* L. (AD2)—have been domesticated, whereas five—*G. tomentosum* Nutall ex Seemann (AD3; DeJoode and Wendel 1992), *G. mustelinum* Miers ex Watt (AD4; Wendel *et al.* 1994), *G. darwinii* Watt (AD5; Wendel and Percy 1990), *G. ekmanianum* Wittmack (AD6; Krapovickas and Seijo 2008; Grover *et al.* 2015b), and *Gossypium* sp. nov. (Wendel and Grover 2015)—are wild. In the most recent phylogenetic relationships among tetraploid cotton species, *G. mustelinum* comprises one branch of the earliest split following allopolyploid formation; *G. hirsutum* was sister to the recently recognized tetraploid *G. ekmanianum*, and they formed a clade sister to *G. tomentosum*; *G. barbadense* was sister to *G. darwinii*, and these two species formed a clade that was sister to the *G. ekmanianum*–*G. hirsutum*–*G. tomentosum* clade (Grover *et al.* 2015b).

The transmission genetics of crosses between the two cultivated tetraploids, *G. hirsutum* and *G. barbadense*, have been investigated in detail, and many interspecific *G. hirsutum* × *G. barbadense* genetic maps have been developed based on different molecular marker types including restriction fragment length polymorphisms (RFLPs), amplified fragment length polymorphisms (AFLPs), random amplified polymorphic DNA (RAPDs), simple sequence repeats (SSRs), and others (Reinisch *et al.* 1994; Rong *et al.* 2004; Lacape *et al.* 2003, 2009; Nguyen *et al.* 2004; Song *et al.* 2005; Park *et al.* 2005; Han *et al.* 2004, 2006; Lin *et al.* 2005; Yu *et al.* 2007, 2011, 2012; He *et al.* 2007; Guo *et al.* 2007, 2008; Blenda *et al.* 2012; Shi *et al.* 2015).

Recent cotton genome sequencing, including the A-genome of *G. arboreum* (Li *et al.* 2014), the D-genome of *G. raimondii* (Paterson *et al.* 2012; K. Wang *et al.* 2012), and the tetraploid AD-genome of *G. hirsutum* (Zhang *et al.* 2015; Li *et al.* 2015), facilitates the construction of high-density maps and comparison between genetic maps and genome sequences. Wang *et al.* (2013) constructed a cotton map comprising 48,958 loci that were aligned to both a consensus genetic map and a reference genome sequence; Hulse-Kemp *et al.* (2015) constructed two high-density genetic maps containing 22,829 SNPs for two F_2_ mapping populations, and 3533 SNP markers co-occurred in both maps.

It would be a valuable research tool to have maps among the tetraploid cottons crossed in all possible combinations as well as maps of intraspecific crosses for each tetraploid. For *G. tomentosum*, a wild tetraploid species that is closely related to *G. hirsutum* (Grover *et al.* 2015a), Waghmare *et al.* (2005) described the first *G. hirsutum* by *G. tomentosum* (HT) map comprising 589 loci based on RFLP markers. Zhang *et al.* (2011) further exploited QTL alleles for improved fiber quality from *G. tomentosum* based on advanced-backcross populations derived from the same cross. Hou *et al.* (2013) constructed an SSR-based HT genetic map consisting of 1204 loci, with a mean density of 2.76 cM per locus. For *G. darwinii*, the tetraploid cotton species most closely related to *G. barbadense* (Wendel and Percy 1990; Grover *et al.* 2015a), B. Wang *et al.* (2012) performed QTL mapping of fiber quality in introgression lines derived from *G. hirsutum* × *G. darwinii*. Chen *et al.* (2015) constructed an interspecific high-density linkage map using an F_2_ population of *G. hirsutum* × *G. darwinii*, which consists of 2763 SSR markers with an average interlocus distance of 1.5 cM.

Little is known about transmission genetics and genome organization in *G. mustelinum*, which is isolated as one branch of the earliest split following allopolyploid formation, and is genetically farthest from *G. hirsutum* (Grover *et al.* 2015a). To reveal the basic transmission genetics in crosses between *G. mustelinum* and cultivated cotton, and build information useful to extract agriculturally valuable alleles from *G. mustelinum*, a primary *G. hirsutum* × *G. mustelinum* (HM) genetic map was constructed and compared with those involving *G. hirsutum* crossed with *G. barbadense*, *G. tomentosum*, and *G. darwinii*, respectively. Colinearity between our HM map and the tetraploid cotton genome was also investigated. To provide an important demonstration of the usefulness of the map, QTL mapping of fiber elongation that differentiates the parental lines was performed in advanced backcross populations derived from the same parents.

## Materials and Methods

### Plant materials

An interspecific F_2_ population comprising 92 plants was developed from a cross between *G. hirsutum* (PD94042) and *G. mustelinum* (AD4-8). PD94042 is a public cotton (*G. hirsutum*) germplasm line that combines high yield potential and improved fiber maturity developed at the Pee Dee Research and Education Center situated in Florence and Darlington counties, SC (May 1999). Plants of the F_2_ population were grown in a greenhouse in Tifton, GA.

Advanced backcross populations were developed by first crossing *G. hirsutum* acc. PD94042 and *G. mustelinum* (AD4-8), then independently backcrossing F_1_ plants to the *G. hirsutum* parent for three cycles. BC_3_F_1_ plants were selfed to generate BC_3_F_2_ families (Wang *et al.* 2016), and 12 BC_3_F_2:3_ and BC_3_F_2:4_ families of 130–160 lines per family (totally 1826 lines with average population size of 152) were planted in 2008 and 2009 in Tifton, GA. Fiber elongation data were collected from two random replicate plots and genetically mapped. All cultural practices followed standard recommendations for Georgia cotton production as described in Wang *et al.* (2016). Fiber elongation was tested by using a High-Volume Precision Instrument (HVI; Zellweger-Uster, Knoxville, TN) in the Cotton Incorporated Textile Services Laboratory (Cary, NC).

### Molecular and morphological markers

The majority of molecular markers utilized here were SSR markers. Most of them were selected from an interspecific *G. hirsutum* by *G. barbadense* map (Guo *et al.* 2007), and the marker sequences were downloaded from the Cotton Marker Database (CMD, http://www.cottonmarker.org). RFLP and sequence-tagged sites (STS), which were largely sampled from published HB (Rong *et al.* 2004) and HT maps (Waghmare *et al.* 2005), were also used in this experiment. In addition, root-related genes in *Arabidopsis* were identified from The *Arabidopsis* Information Resource (TAIR, http://www.arabidopsis.org), and their homologous cotton expressed sequence tags (ESTs) were obtained through “basic local alignment search tool” (BLAST) searches of an online cotton EST database (http://www.agcol.arizona.edu/cgi-bin/pave/Cotton/index.cgi). Eighteen pairs of EST-SSR primers were designed as described by Han *et al.* (2006), and designated here using UGT (the University of Georgia, Tifton Campus) as a prefix (Table 1). Enzyme digestion was utilized on the PCR products of UGT primer without polymorphism, and used as cleaved amplified length polymorphisms (CAPs) described by Konieczny and Ausubel (1993). Three CAPs were analyzed, namely CAPs0005, amplification products of UGT0005 digested by *Hin*fΙ; CAPs0010, amplification products of UGT0010 digested by *Hae*III; and CAPs0011, amplification products of UGT0011 digested by *Hha*Ι. Three morphological markers, namely anther color (yellow or cream, *P1* gene), petal color (yellow or cream, *Y1* gene), and petal spot (presence or absence, *R2* gene; Kohel and Richmond 1971) were also investigated.

**Table 1:** Targeted *Arabidopsis* root-related gene homologs

Primer Name	*Arabidopsis* Gene	Homologous Cotton EST	Score	BLAST E Value	Sense Primer (5′–3′)	Antisense Primer (5′–3′)
UGT0001	AT2G28350	GR__Ea18I12.f	72	5.00E-11	TATCTTTATCCGATCTCCATC	CACTGCCATCTAACGAACTA
UGT0002	AT3G62980.1	GR__Ea24P15.f	168	3.00E-40	TTGTTGCCGTATCTTTGGGTTGT	CCCGGAAAGCACATGATGTAGTC
UGT0003	AT1G55020	GH_CHX21G20.x	88	1.00E-15	CTTATGCGTCTCGAACCATC	CCAACTGCCATATTGAACCT
UGT0004	AT1G79840	GH_BNL1AF336277.x	96	4.00E-18	GCGAGTGCGAGTATGGAGGTG	TCTGATTTGGTCGGCGGTGT
UGT0005	AT1G13290	GA__Ed0105E03.f	80	1.00E-13	GGTGATGATTCTTCTGGGTG	TCTTAACATTCCGGTTGGTT
UGT0006	AT1G23080	GR__Eb05J17.f	88	8.00E-16	TTTCAGACGCAAGCAGCAGG	CAATCCAAGAGCGAAGAGCA
UGT0007	AT1G48410	GR__Ea18B04.f	256	2.00E-66	CCGAAGAGCAACTGGACATA	ACAACAAAGGTAACAGGAGG
UGT0008	AT1G73590	GR__Eb02O18.f	100	2.00E-19	TGAAGATGGTGGTGGTAAGG	CTCGTTGGTGGCATGGTTTT
UGT0009	AT2G24790	GH_SUO1AJ513465.x	109	1.00E-22	CATTGTTGGGTGGGATTAT	CCGTTACGCCTCCAGAAAA
UGT0010	AT2G33880	GH_SCW21H1.x	111	4.00E-23	TTTTGAGGTTGCTGCTGAT	AAGTAGGTGATGCCAATGTG
UGT0011	AT2G44900	GR__Eb0035B16.f	206	2.00E-51	CTAGCCCAATCCTGTTCAA	GAGCGAGCAAGAGCAATC
UGT0012	AT3G04630	GH_ECOT8CE08T3_056.x	80	1.00E-13	AAGGGATCAGAGCCAAAC	TGGGAGGTCCTTCATAGTAGA
UGT0013	AT3G16785	GR__Ea12N20.f	232	4.00E-59	TGCTTATTGCTCCCTCAT	TAGCCACAGGACCGTGAT
UGT0014	AT3G17600	GR__Eb03P09.f	68	3.00E-10	TATTCCTCCCTCCTACTCG	AACCCTAAACGCAACTCC
UGT0015	AT3G50060	GA__Ed0080F06.f	82	3.00E-14	AACCACCGTTGTAACCTTCC	CTTTGATCCGATCCATATCTTT
UGT0016	AT3G60350	GH_SDL0009016.H02_010601206K.f	204	8.00E-51	GCTGGTGGTGGTATTGAGG	CGAAGAGCGTTACTATGGTTAA
UGT0017	AT4G00730	GH_ON34K17.r	90	9.00E-16	TTGCTATCCTTATGTCCTCCTCT	GTCAACGCTCTTTCGGGTC
UGT0018	AT2G46990	GH_SCW84_F06_038.x	76	9.00E-13	GGGATTGGATGATGGTTGGT	AAGACTGGTGCTTGTTACTC

### DNA extraction, RFLP protocol, PCR amplification, and electrophoresis

DNA extractions followed the protocols established by Paterson *et al.* (1993). RFLP analysis was performed as described by Reinisch *et al.* (1994). SSR and STS-PCR amplifications were performed using a Peltier Thermal Cycler-225 (MJ Research) as described by Zhang *et al.* (2002) with modifications: predenaturation at 94° for 3 min; 30 cycles of 40 sec denaturation at 94°, 45 sec annealing at 57°, and 1 min extension at 72°; 7 min extension at 72°; and finally a 10° hold. Enzyme digestion of CAPs was performed as described by Chee *et al.* (2004) after the UGT primers were amplified. Polyacrylamide gel electrophoresis of SSR, STS, and CAPs was performed as described by Zhang *et al.* (2002).

### Construction of a genetic linkage map

A genetic linkage map was constructed using MAPMAKER/Exp Version 3.0 (Lander *et al.* 1987) software, in which the Kosambi centiMorgan function and threshold LOD = 5.0 were used. Linkages at distances of greater than 35 Kosambi cM were considered to be nonsignificant. Assignment of linkage groups to subgenomes and chromosomes was made based on the information from framework markers on the published maps (Reinisch *et al.* 1994; Nguyen *et al.* 2004; Rong *et al.* 2004; Park *et al.* 2005; Guo *et al.* 2007).

### Sequence homology between markers and G. hirsutum genome

The reference genome of the tetraploid species *G. hirsutum* (http://mascotton.njau.edu.cn; Zhang *et al.* 2015) was used in this study. Markers mapped on the HM map with available sequences were aligned to the scaffold sequences using the BLASTN algorithm with an e-value cutoff of 1e-5 and an identity percentage cutoff of 90%. All hits separated by distances of 5 kb or less were assembled into single loci, and we retained up to the top 10 matching loci with 95% length coverage of the original marker sequence for each marker.

### Application of the HM map in QTL mapping of fiber elongation

The genome structure of the BC_3_F_1_ individuals was evaluated based on genotyping the DNA of BC_3_F_1_ plants with 218 SSR markers, which were approximately evenly distributed on the HM map. The markers with introgression from *G. mustelinum* in the BC_3_F_1_ were then used to screen the entire BC_3_F_2_ family (Wang *et al.* 2016).

The mixed model-based composite interval mapping (MCIM) of QTL Network V2.1 (Yang *et al.* 2008) was used to analyze main-effect additive QTL with the critical F value of MCIM being calculated based on 1000 permutation tests. QTL effects were estimated using the Monte Carlo Markov Chain method with 20,000 Gibbs sampler iterations, and candidate interval selection. Gibbs sample size was set to the default value. Window size and walk speed were set at 10 cM and 1 cM, respectively. The threshold for significance was set at *P* = 0.001 to claim a putative QTL. Since QTL for fiber elongation were mapped in 21 BC_3_F_2_ populations in our previous report (Wang *et al.* 2016), here QTL mapping was performed in two additional generations/environments, namely BC_3_F_2:3_ and BC_3_F_2:4_, and also in joint analysis of the three generations/environments (BC_3_F_2_, BC_3_F_2:3_, and BC_3_F_2:4_) considering the environmental effects.

### Data availability

The sequences of microsatellite markers for this project are available at both CottonGen (https://www.cottongen.org/) and Cotton Marker Database (CMD, http://www.cottonmarker.org); the sequences of RFLP probes for this project are available at CottonGen (https://www.cottongen.org/). Detailed comparison between HM map with HT, HB, and HD maps are available in Supplemental Material, Figure S1. The raw marker data used to construct the HM map are available in File S1, and the genotype data and phenotype data used to map QTL of fiber elongation are available in File S2.

## Results

### General information about the genetic map

**Figure 1:**
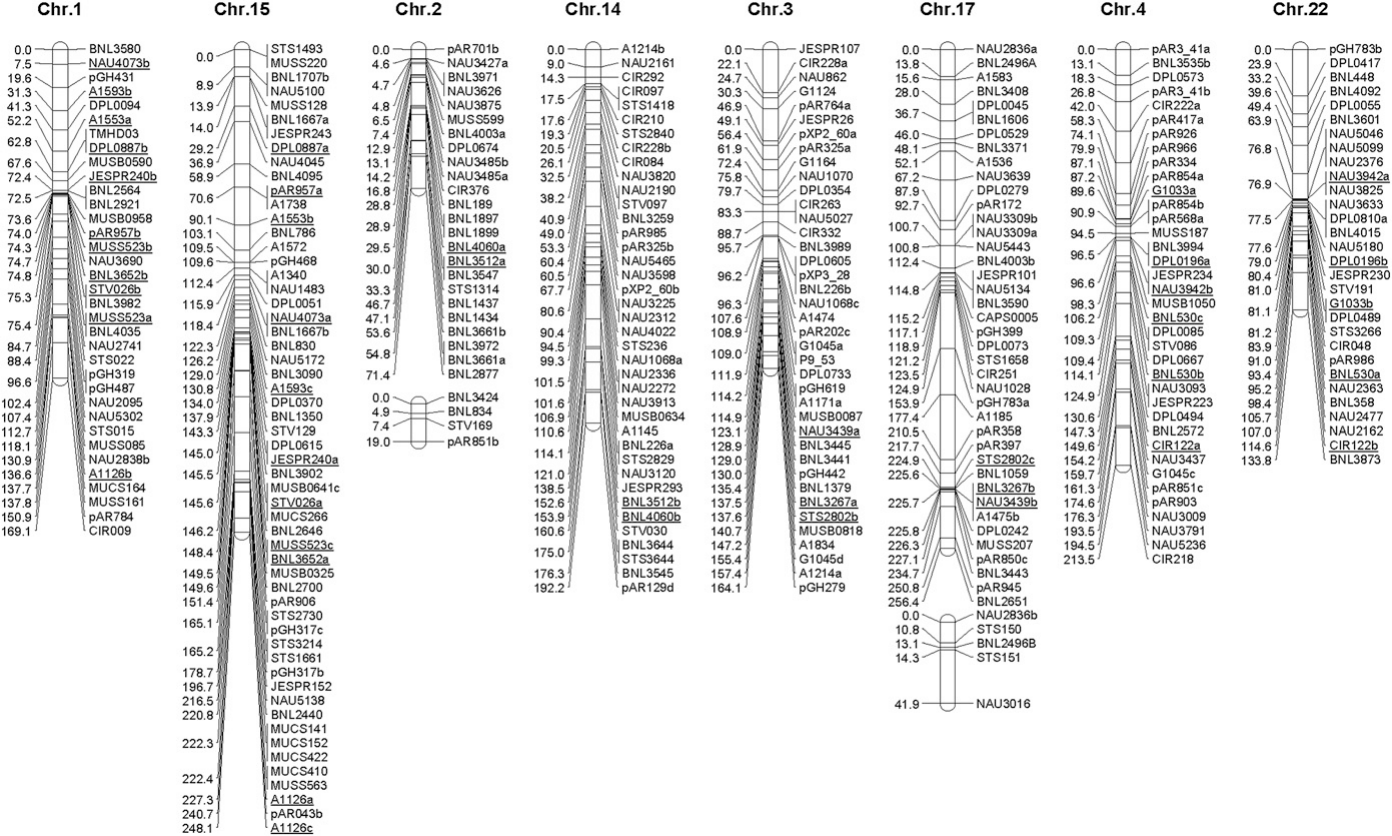

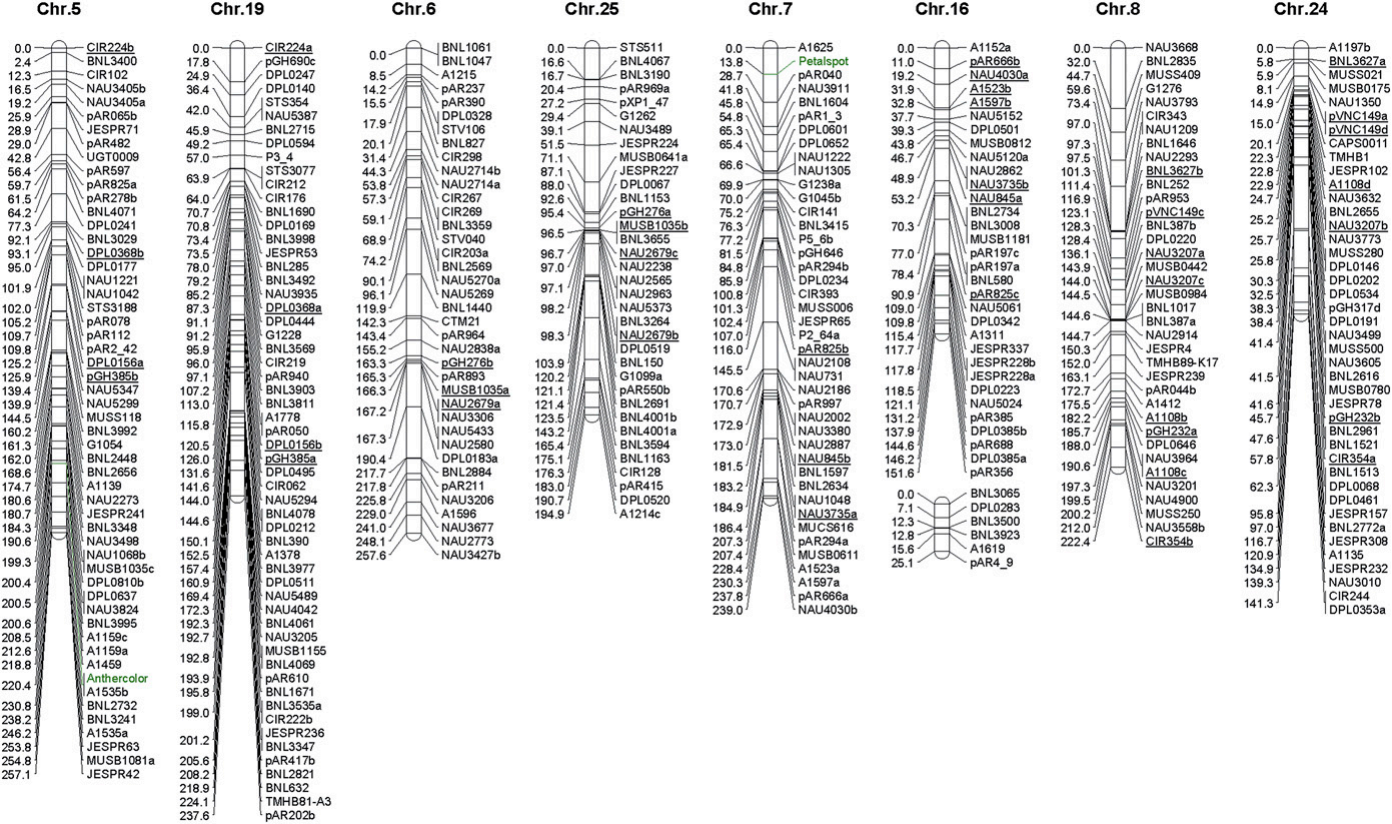

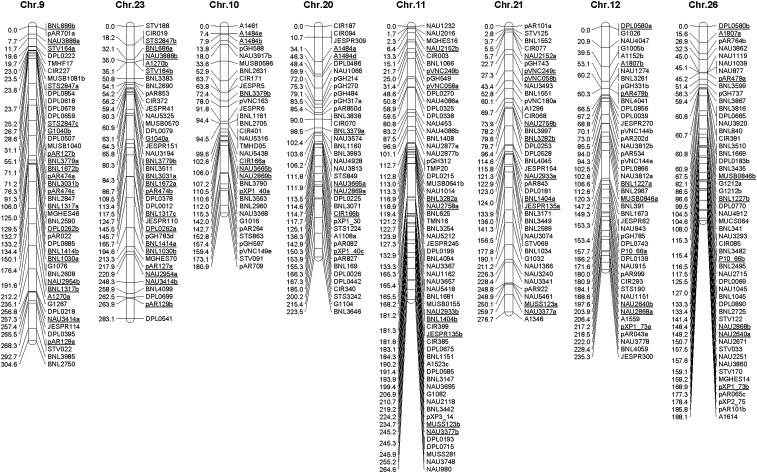

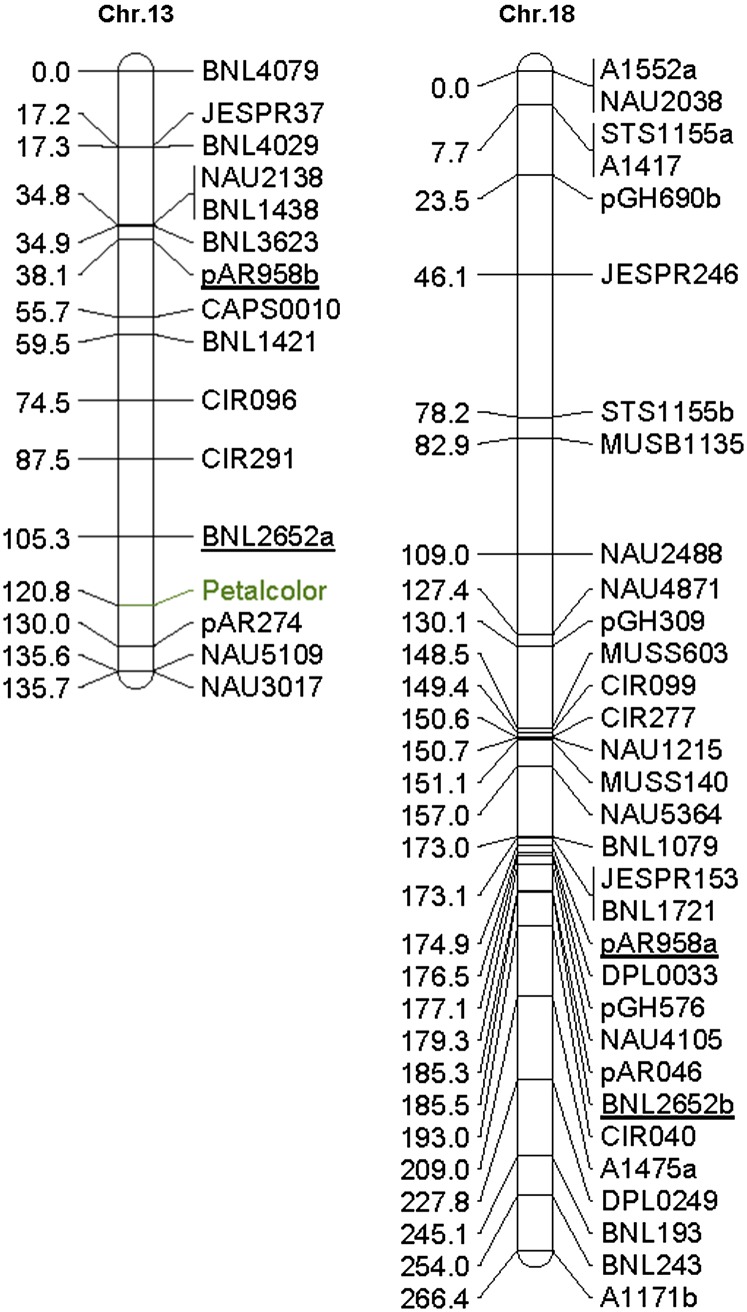
*Gossypium hirsutum* × *G. mustelinum* (HM) genetic map. The 26 chromosomes are arranged according to the 13 homeologous pairs of A (Chr.1–Chr.13) and D (Chr.14–Chr.26) chromosomes, *i.e.*, Chr.1 (At genome) is followed by its homeologous Chr.15 (Dt genome), etc. Underlined loci are duplicated loci. Map distances are given in centiMorgans (cM). Locus names are as described in *Materials and Methods*.

A total of 690 polymorphic SSR primers, 201 cDNA and genomic DNA probes, 29 STS primers, three CAPs and three morphological markers were analyzed in the F_2_ progeny (the raw marker data are listed in File S1). Among the 926 markers mapped, a total of 163 detected multiple loci, namely 129 detected two, 25 detected three, seven detected four, and two detected five loci. In total, 1134 loci were generated, of which 988 (87.1%) did not deviate significantly from—and 146 (12.9%) deviated from—Mendelian 1:2:1 (for codominant) and 3:1 (for dominant) inheritance ratios. A total of 674 loci were codominant, whereas 228 were dominant for the *G. hirsutum* allele, and 232 were dominant for the *G. mustelinum* allele. The HM map constructed here comprises 1055 loci on 26 chromosomes (Figure 1 and Table 2), with total recombinational length of 5595 cM, within the 3500–5700 cM range of *G. hirsutum* by *G. barbadense* (HB) maps (Lacape *et al.* 2009).

**Table 2:** General information of chromosomes in the genetic map of *G. hirsutum* and *G. mustelinum*

Chromosome	Marker No.	Size (cM)	Average Distance (cM)
Chr.1	35	169.1	4.8
Chr.2	28	90.4	3.2
Chr.3	39	164.1	4.2
Chr.4	37	213.5	5.8
Chr.5	54	257.1	4.8
Chr.6	38	257.6	6.8
Chr.7	42	239.0	5.7
Chr.8	37	222.4	6.0
Chr.9	46	304.6	6.6
Chr.10	33	180.9	5.5
Chr.11	60	264.6	4.4
Chr.12	45	235.3	5.2
Chr.13	16	135.7	8.5
At-Total	510	2734.3	5.4
Chr.14	39	192.2	4.9
Chr.15	56	248.1	4.4
Chr.16	38	176.7	4.7
Chr.17	45	298.3	6.6
Chr.18	32	266.4	8.3
Chr.19	57	237.6	4.2
Chr.20	39	223.5	5.7
Chr.21	40	276.7	6.9
Chr.22	30	133.8	4.5
Chr.23	39	283.1	7.3
Chr.24	42	141.3	3.4
Chr.25	35	194.9	5.6
Chr.26	53	188.1	3.5
Dt-total	545	2860.7	5.2
Total	1055	5595.0	5.3

### Characteristics of the HM map

Among the 1055 loci mapped on chromosomes, about 6.9% more markers were detected in the D than the A subgenome (545 *vs.* 510), and the recombinational length was about 4.6% larger in the D than the A subgenome (2860.7 *vs.* 2734.3 cM). The average recombination distance between consecutive loci was 5.3 cM, whereas the density of markers along chromosomes ranged from 3.2 cM (Chr.2) to 8.5 cM (Chr.13). The largest gap between two adjacent loci was 33.5 cM (Chr.24). The overall average recombination distance in the two subgenomes was similar (5.4 cM in At *vs.* 5.2 cM in Dt). Significant variation in chromosome length was observed, ranging from 90.4 cM (Chr.2) to 304.6 cM (Chr.9). Seven At chromosomes were longer than homeologous Dt chromosomes, whereas six At chromosomes were shorter than Dt chromosomes (Table 2).

Tetraploid cotton containing At and Dt subgenomes was derived from a naturally occurring cross between two diploids with A and D genomes about 1–2 million yr ago (Wendel and Cronn 2003). The distributions of duplicate loci (Figure 1) were generally consistent with the homeologous relationships among chromosomes that are well established in the HB, HT, and HD maps (Rong *et al.* 2004; Waghmare *et al.* 2005; Guo *et al.* 2007; Chen *et al.* 2015). Many loci were also duplicated on chromosomes that were nonhomeologous (Figure 1), perhaps reflecting single-gene duplication, or earlier genome duplication events (Paterson *et al.* 2012).

Nonrandom patterns of DNA marker distribution provided clues regarding important features of cotton genome organization. Unlike the duplicate loci on homeologous chromosomes, nonhomeologous duplicate loci were scattered over many chromosomes. For example, 13 markers on Chr.5 detected duplicate loci, with four markers having duplicate loci on homeologous Chr.19, three on Chr.5 itself, and the other six scattered on nine other chromosomes. The intrasubgenomic duplications have been proposed as supporting the ancient chromosomal duplication hypothesis (paleo-polyploidization) predating divergence of modern *Gossypium* diploid genomes, that has now been shown to have been a 5–6 × multiplication of ancestral chromosomes (Paterson *et al.* 2012).

### Chromosome structural differences between HM, HB, HT, and HD maps

**Figure 2:**
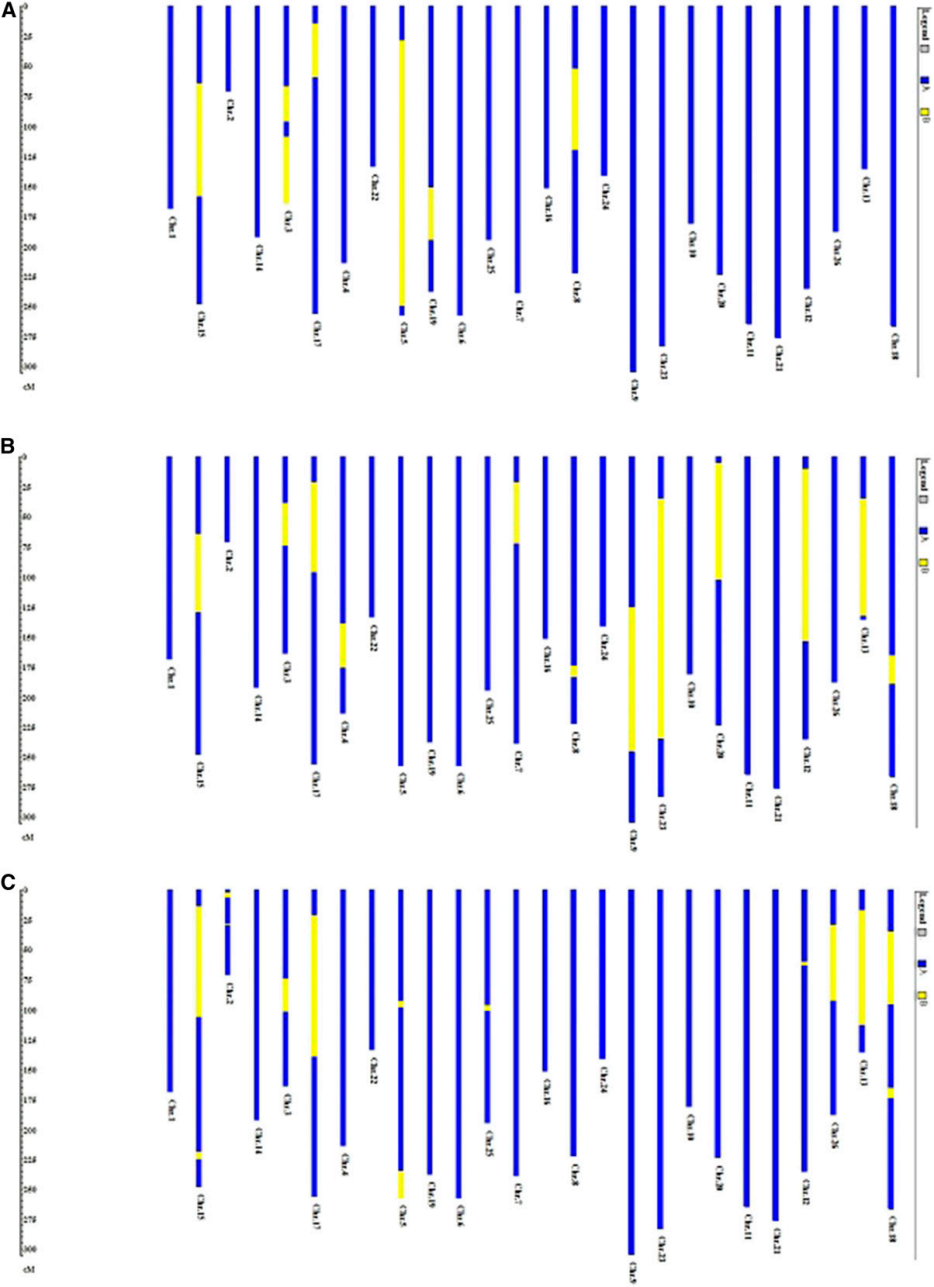

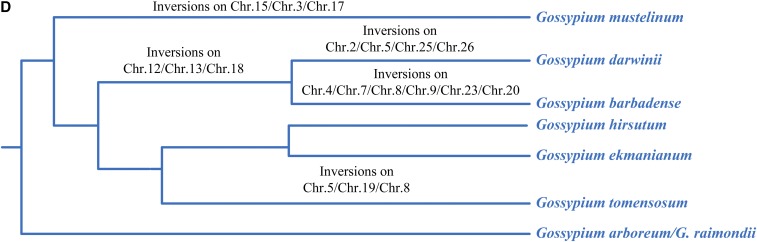
Inversions found in HT (A), HB (B), and HD (C) maps compared to the HM map. D indicates when each inversion occurred during tetraploid cotton diversification based on the phylogeny proposed by Grover *et al.* (2015a).

Based on alignment of common DNA markers, our HM map was compared to HT (Waghmare *et al.* 2005), HB (HBr, Rong *et al.* 2004; HBg, Guo *et al.* 2007), and HD (Chen *et al.* 2015) maps, respectively. The inversions found between HM and HT, HM and HB, HM and HD maps are shown in Figure 2 and Figure S1. A summary of the detailed comparisons of HM with maps of different species follows.

A possible inversion was found on Chr.15 when comparing HM with HT, HB, and HD (Figure 2 and Figure S1). In HM and HT, the inversion was detected based on seven anchor markers, namely pAR957, A1738, A1553, pGH468, A1340, A1593, and pAR906, with the affected regions spanning 80.8 cM in HM and 75.6 cM in HT; two of these anchor markers, pAR957 and A1738 cosegregated in HM, but were 13.8 cM apart in HT, consistent with a possible inversion. In HM and HB, the inversion was found based on six anchor markers pAR957, A1738, A1553, pGH468, A1340, and BNL3090, with the affected regions spanning 58.4 cM in HM and 101.8 cM in HBr; the two anchor markers pAR957 and A1738 cosegregated in HM, but they were 8.6 cM apart in HBr, consistent with a possible inversion. In HM and HD, the inversion was divided into two sections, the first based on four anchor markers BNL1667a, JESPR243, NAU4045, and BNL786, with the affected regions spanning 89.1 cM in HM and 24.9 cM in HD, and the second based on another four anchor markers, namely BNL2440, MUCS422, MUCS410, and MUSS563 (Figure 2 and Figure S1).

On Chr.3, two anchor markers G1164 and pXP3_28 cosegregated in HT, but were 23.8 cM apart in HM, consistent with a possible inversion; a second possible inversion was found based on five anchor markers, namely pGH279, P9_53, pGH619, A1171, and A1834, with the affected regions spanning 55.1 cM in HM *vs.* 44.4 cM in HT. Based on three anchor markers (pAR764, pXP2_60, and G1164), an inversion distinguished HM Chr.3 from HBr; the affected regions spanned 25.5 cM in HM and 19.4 cM in HBr. HM Chr.3 was differentiated from HD by an inversion based on five anchor markers (NAU1070, NAU5027, BNL3989, BNL226b, and NAU1068c), with the affected regions spanning 20.5 cM in HM compared to 14.8 cM in HD (Figure 2 and Figure S1).

Based on two anchor markers A1583 and A1536, an inversion appears to distinguish HM Chr.17 from HT; the affected regions spanned 36.5 cM in HM compared to only 8.5 cM in HT. An overlapped inversion was found on Chr.17 between HM and HBr based on three anchor markers BNL3408, A1536, and pAR172; the affected regions spanned 64.7 cM in HM whereas only 3.6 cM in HBr. Based on five anchor markers BNL3408, DPL0529, DPL0279, BNL4003b, and NAU1028, an inversion distinguished HM Chr.17 from HD, with the affected regions spanning 96.9 cM in HM compared to 31.2 cM in HD (Figure 2 and Figure S1).

An inversion was indicated on Chr.5 based on eight anchor markers between HM and HT, namely A1159, A1459, A1535, pAR482, pAR597, pAR825, pAR112, and pAR2_42; the affected regions spanned 217.2 cM in HM *vs.* only 109.7 cM in HT. Two inversions were detected on Chr.5 between HM and HD. The first inversion was based on two anchor markers, DPL0368b and DPL0177; the second inversion was based on two anchor markers BNL3241 and JESPR42, with the affected regions spanning 18.9 cM in HM comparing to only 14.4 cM in HD (Figure 2 and Figure S1).

Based on two anchor markers, G1276 and pAR953, an inversion distinguished HM Chr.8 from HT, and the affected regions spanned 57.3 cM in HM and 33.7 cM in HT. An inversion was also found on Chr.8 between HM and HBr based on two anchor markers A1412 and A1108, with the affected regions spanning 6.7 cM in HM comparing to 3.6 cM in HBr (Figure 2 and Figure S1).

Three overlapped inversions were found when comparing HM with both HBr and HBg (Figure 2 and Figure S1). On Chr.9, *G. mustelinum* was differentiated from *G. barbadense* by an inversion based on four anchor markers BNL2590, BNL1030, A1270b, and G1267b; the affected regions spanned 110.1 cM in HM comparing to 54.5 cM in HBr. When compared to HBg, this inversion was also found on Chr.9 based on three anchor markers BNL2590, BNL1414, and BNL1030; the affected regions spanned 25.1 cM in HM compared to 9.9 cM in HBg. On Chr.23, based on four anchor markers A1270, pGH783b, BNL3031, and pAR474, an inversion distinguished HM Chr.23 from HBr, with affected regions spanning 110.7 cM in HM, whereas only 29.2 cM in HBr; when compared to HBg, an overlapped inversion was detected based on three anchor markers JESPR110, BNL1414, and NAU2954, with the affected regions spanning 96.2 cM in HM, whereas only 8.7 cM in HBg. The third overlapped inversion was found on Chr.13. Based on three anchor markers pAR958, BNL2652, and pAR274, an inversion distinguished HM Chr.13 from HBr, with the affected regions spanning 91.9 cM in HM *vs.* 27.1 cM in HBr; an overlapped inversion was found on Chr.13 of HBg based on three anchor markers BNL1438, BNL1421, and BNL2652; the affected regions spanned 70.5 cM in HM compared to 9.7 cM in HBg. This third inversion was also found between HM and HD on Chr.13 based on four anchor markers, namely BNL4029, BNL1438, BNL1421, and BNL2652a, with the affected regions spanning 88 cM in HM but only 22.5 cM in HD.

A terminal inversion was found on Chr.12 based on six anchor markers NAU4047, NAU1274, BNL3261, NAU3812, BNL1673, and NAU943, with the affected regions spanning 132.4 cM in HM and 44.4 cM in HBg. An overlapped terminal inversion was found on Chr.12 between HM and HD based on two anchor markers BNL3261 and BNL4041, with the affected regions spanning only 0.1 cM in HM but 2.4 cM in HD (Figure 2 and Figure S1).

Based on five anchor markers BNL1079, BNL1721, pGH576, pAR046, and BNL2652, HM Chr.18 differed from HBr Chr.18 by an inversion; the affected regions spanned 12.5 cM in HM and 42.1 cM in HBr. An overlapped inversion was found on Chr.18 between HM and HD based on two anchor markers BNL1079 and BNL1721, with the affected regions spanning only 0.1 cM in HM but 10.6 cM in HD; another inversion was found on Chr.18 between HM and HD based on two anchor markers, JESPR246 and MUSB1135, with the affected regions spanning 36.8 cM in HM but only 5.8 cM in HD (Figure 2 and Figure S1).

Some cases of inversions between HM and only one other mapped tetraploid species (HT, HB, and HD) were also detected (Figure 2 and Figure S1). Two inversions were detected on Chr.2 between HM and HD; the first inversion was based on four anchor markers, namely NAU3427a, BNL3971, AU3875, and MUSS599, with the affected regions spanning 1.9 cM in HM and 16 cM in HD; the second inversion was based on two anchor markers, BNL1897 and BNL3512a, with similar distance between these two loci in both maps. Based on three anchor markers BNL2572, G1045, and pAR903, an inversion was found on Chr.4 between HM and HBr, with the affected regions spanning 27.3 cM in HM and 29 cM in HBr. An inversion was found based on two anchor markers A1378 and pAR610 on Chr.19 between HM and HT; the affected regions spanned 41.4 cM in HM but 19.9 cM in HT. Based on three anchor markers NAU2679c, NAU2565 and DPL0519, an inversion appears to distinguish HM Chr.25 from HD, with the affected regions spanning 1.6 cM in HM *vs.* 5 cM in HD. An inversion was found on Chr.7 between HM and HBr based on two anchor markers pAR040 and G1045; the affected regions spanned 41.3 cM in HM, whereas only 0.5 cM in HBr. A terminal inversion was found on Chr.20 between HM and HBg based on three anchor markers CIR094, NAU1066, and NAU3574 (Figure S1), with the affected regions spanning 91.7 cM in HM and 20.6 cM in HBg. Based on nine anchor markers, namely NAU3862, NAU1039, BNL3867, NAU3920, BNL840, BNL3510, DPL0183b, MUSB0846b, and BNL1227b, a terminal inversion distinguished HM Chr.26 from HD, with the affected regions spanning 54.3 cM in HM and 42.1 cM in HD.

### Homology of the HM genetic map with the tetraploid cotton genome

**Figure 3:**
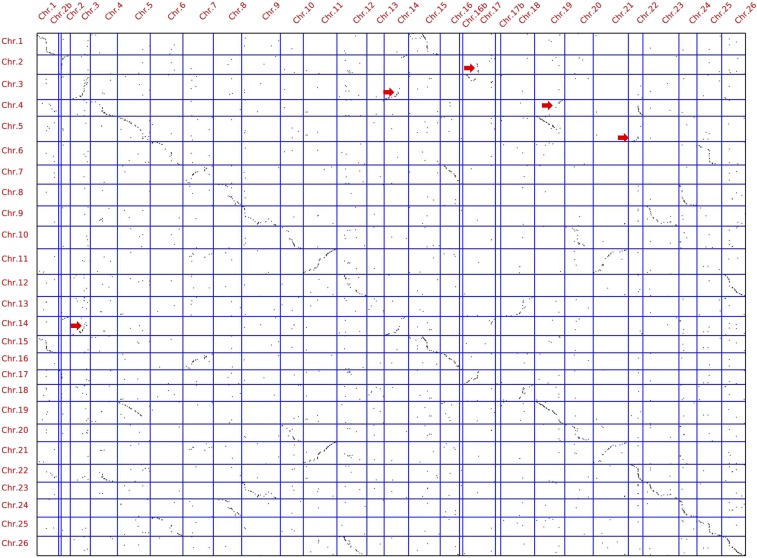
Dotplots of the syntenic positions of molecular markers in the HM genetic linkage map *vs.* the *G. hirsutum* reference genome. The *G. hirsutum* reference genome chromosomes are shown on the *y*-axis and the HM genetic linkage map chromosomes are shown on the *x*-axis. Red arrows indicate translocation events relative to *G. hirsutum* reference genome.

The result of colinearity analysis between the linkage map and genome sequences of *G. hirsutum* (Zhang *et al.* 2015) is shown with dotplots in Figure 3. Most marker positions correspond closely between the two maps, and translocations involving Chr. 2 with Chr.3 and Chr.4 with Chr.5 were identified (Figure 3), whereas some discrepancies existed.

### Assignment of morphological markers and root-related cotton ESTs to chromosomes

Three morphological traits were investigated in the F_2_ population of *G. hirsutum* × *G. mustelinum*, namely anther color (*P1*), petal color (*Y1*), and petal spot (*R2*). The chi-squared values for yellow *vs.* cream anther; yellow *vs.* cream petal; and presence *vs.* absence of petal spot were 0.90, 0.44, and 0.73, respectively; each does not deviate significantly from the Mendelian segregation ratio for a single gene (3:1) (χ_c_^2^ *<* χ_0.05,1_^2^ = 3.84). The three traits were used as morphological markers to construct the genetic map. Anther color was associated with the interval between A1459 and A1535b on Chr.5; petal color was associated with the interval between BNL2652a and PAR274 on Chr.13; petal spot was associated with the interval between A1625 and pAR040 on Chr.7 in our research (Figure 1).

UGT primers were synthesized according to cotton ESTs homologous to root-related *Arabidopsis* genes, of which UGT0009 was mapped on Chr.5. Three CAPs were also mapped, namely CAPs0005 on Chr.17, CAPs0010 on Chr.13, and CAPs0011 on Chr.24. According to TAIR, the *Arabidopsis* genes homologous to these cotton ESTs are involved in root development; the inclusion of these genes may help detect cotton root QTL and build on the connections between cotton and *Arabidopsis* genes.

### Phenotypic performance and QTL mapping of fiber elongation in advanced-backcross populations

**Figure 4:**
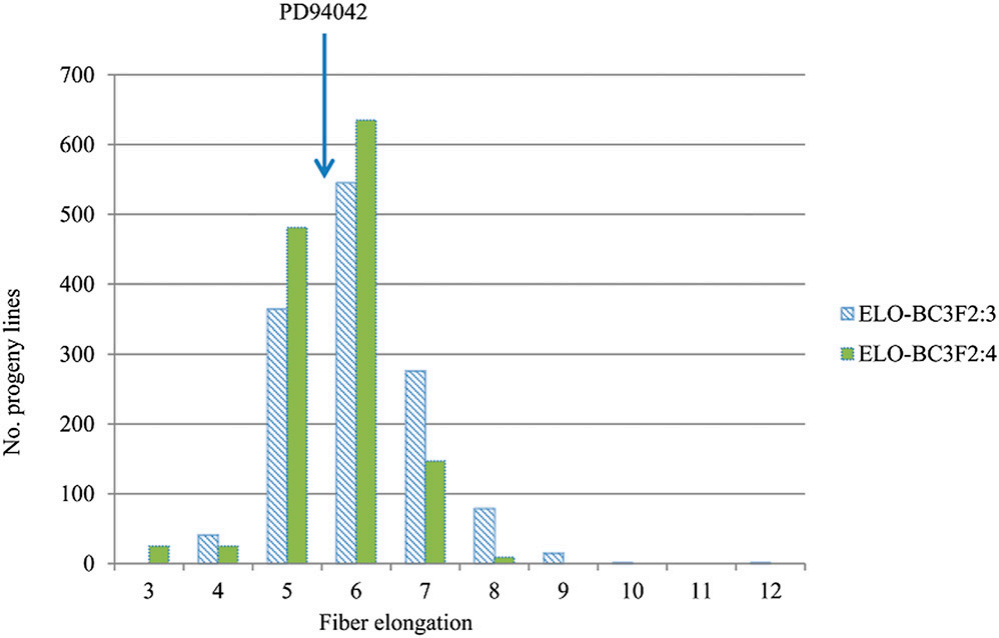
Frequency distribution of fiber elongation in the BC_3_F_2:3_/BC_3_F_2:4_ progeny lines.

The distribution of fiber elongation in the BC_3_F_2:3_ and BC_3_F_2:4_ generations is shown in Figure 4. The 12 families as a whole were normally distributed for fiber elongation in both generations. Although *G. mustelinum* does not produce spinnable fiber, many BC_3_F_2:3_ and BC_3_F_2:4_ lines have higher fiber elongation than the *G. hirsutum* parent.

By assuming that QTL with common flanking marker(s) and additive effects from the same direction represent single QTL, a total of 14 fiber elongation QTL (*P* < 0.001) were detected (Table 3), and six of them were also detected in BC_3_F_2_ generations (Wang *et al.* 2016). These QTL were mapped to 12 chromosomes; eight on six chromosomes from the A-subgenome, and six on six D-subgenome chromosomes. QTL were detected in 10 of the 12 families, with one to six per family, and a maximum of six QTL detected in family POP16. The phenotypic variation explained per QTL ranged from 1.24 to 19.41%, with an average of 10.14%. Eight QTL could be detected at least twice in different families or across different environments; notably, the QTL *qELO-19-1* could be detected in three environments and also the joint analysis. Ten of the 14 QTL had positive additive effects, where alleles from *G. hirsutum* increased fiber elongation, which was consistent with the parental phenotypes. The remaining four of the 14 QTL had negative additive effects, where alleles from *G. mustelinum* increased fiber elongation (Table 3).

**Table 3:** Estimates of additive effects of quantitative trait loci for fiber elongation across different environments by mixed linear model

QTL*^a^*	Environment*^b^*	Family	Interval	A*^c^*	*P*-Value	h^2(a)(%)*^d^*
*qELO-1-1*	Joint	POP15	MUSS523b-NAU2095	−0.23	0.000000	3.71
*qELO-2-1**^a^*	BC_3_F_2:3_	POP17	BNL1434-BNL3972	0.46	0.000000	18.83
	BC_3_F_2:4_	POP17	BNL1434-BNL3972	0.23	0.000017	13.65
	Joint	POP17	BNL1434-BNL3972	0.43	0.000000	16.86
*qELO-3-1*	BC_3_F_2:4_	POP35	DPL0354-DPL0605	0.26	0.000116	11.57
	Joint	POP16	DPL0354-DPL0605	0.14	0.000801	7.13
*qELO-5-1**^a^*	BC_3_F_2:3_	POP34	BNL3400-CIR102	0.37	0.000053	13.30
	BC_3_F_2:4_	POP34	BNL3400-CIR102	0.31	0.000098	10.91
	Joint	POP34	BNL3400-CIR102	0.33	0.000000	7.93
*qELO-5-2*	BC_3_F_2:3_	POP35	NAU3498-BNL3995	0.49	0.000000	19.41
	BC_3_F_2:4_	POP35	NAU3498-BNL3995	0.24	0.000955	10.96
	Joint	POP35	NAU3498-BNL3995	0.35	0.000000	15.46
*qELO-10-1*	BC_3_F_2:3_	POP35	JESPR6-BNL1161	−0.28	0.000744	5.87
*qELO-11-1**^a^*	BC_3_F_2:3_	POP16	BNL3442-MUSS123b	−0.54	0.000000	14.89
	BC_3_F_2:3_	POP17	BNL3442-MUSS123b	−0.40	0.000002	6.63
	Joint	POP16	BNL3442-MUSS123b	−0.54	0.000000	11.59
	Joint	POP31	BNL3442-MUSS123b	−0.47	0.000007	10.15
	Joint	POP17	MUSS123b-NAU3377b	−0.35	0.000000	2.71
*qELO-11-2*	Joint	POP32	BNL1408-TMP20	0.31	0.000001	8.27
*qELO-18-1*	Joint	POP34	STS1155b-NAU2488	0.30	0.000011	8.20
*qELO-19-1**^a^*	BC_3_F_2:3_	POP15	BNL3811-BNL3977	0.23	0.000157	7.76
	Joint	POP15	BNL3811-BNL3977	0.25	0.000000	7.47
	Joint	POP27	BNL3977-NAU3205	0.37	0.000000	7.36
	Joint	POP17	BNL3977-NAU5489	0.30	0.000000	10.70
	BC_3_F_2:4_	POP16	NAU3205-BNL3535a	0.24	0.000182	14.07
	Joint	POP16	NAU3205-BNL3535a	0.19	0.000034	12.98
*qELO-21-1*	BC_3_F_2:4_	POP16	BNL3171-BNL2589	0.24	0.000427	13.80
*qELO-22-1**^a^*	BC_3_F_2:3_	POP10	DPL0055-NAU2376	0.35	0.000419	10.30
	BC_3_F_2:4_	POP10	DPL0055-NAU2376	0.26	0.000232	11.11
	Joint	POP10	DPL0055-NAU2376	0.34	0.000000	10.71
	Joint	POP11	DPL0055-NAU2376	0.36	0.000000	9.95
*qELO-24-1*	Joint	POP16	NAU3605-DPL0068	0.21	0.000003	5.06
*qELO-26-1**^a^*	Joint	POP16	STV122-NAU3860	−0.27	0.000000	1.24
	Joint	POP34	BNL2725-STV122	−0.37	0.000000	4.21

a Same QTL was detected in BC_3_F_2_ generation (Wang *et al.* 2016).

b Joint = the results were obtained based on combined data of the three generations of BC_3_F_2_, BC_3_F_2:3_, and BC_3_F_2:4_.

c Additive effect of the QTL. A positive number indicates that the alleles from the *G. hirsutum* parent increase trait values; a negative number indicates that the alleles from the *G. mustelinum* parent increase trait values.

d Phenotypic variance explained by additive effects.

## Discussion

### Chromosome structural changes

Two post-polyploidization reciprocal translocations of Chr.4/Chr.5 and Chr.2/Chr.3 were further confirmed by many homologous loci. At-genome chromosomes 4 and 5 have homeologous relationships with two Dt-genome chromosomes (Chr.22 and Chr.19). Nonoverlapping sets of loci on Chr.4 and Chr.5 have counterparts on different regions of Chr.19 and Chr.22 (Figure 1 and Figure 3), consistent with the finding (Brubaker *et al.* 1999) that Chr.4 and Chr.5 have undergone a reciprocal translocation, which is also consistent with results from Rong *et al.* (2004). At-genome chromosomes 2 and 3 have homeologous relationships with Dt-genome chromosomes 14 and 17. Nonoverlapping sets of loci on Chr.2 and Chr.3 have counterparts on different regions of Chr.14 and Chr.17 (Figure 1 and Figure 3), consistent with the results from Guo *et al.* (2007).

Across most pairs of homeologous chromosomes, the linear order of loci was substantially conserved (Figure 1), although some inversions existed. Most locus order differences were due to reversals of neighboring markers explicable by inversions. Many apparent inversions involved only two neighboring loci, and, in our comparatively small mapping population (92 plants), may be due to occasional scoring errors or missing data. However, some putatively orthologous loci mapped to significant different locations and cannot be easily explained by inversion. For example, A1270 mapped to locations of 212.2 cM on Chr.9 and 35 cM on Chr.23, whereas the next common marker NAU3888 was mapped to location of 7.7 cM on Chr.9 but cosegregated with A1270 on Chr.23. It may be ancient duplication together with structural changes, or proximal duplication accompanied by failure to find the true ortholog due to either lack of polymorphism or its deletion, that lead to such differences (Rong *et al.* 2004).

### Mapping of morphological markers

Cotton morphological mutants have been used widely in genetic mapping, and have proven useful in efforts of agronomic improvement in some cases (Kohel and Bird 2002; Ahuja and Dhayal 2007).

Early reports (Harland 1929) proposed that cotton anther color was conditioned by one pair of alleles, *P* and *p*, with yellow dominant to cream. Stephens (1954) proposed that the anther color locus in amphidiploid cotton was located in the A genome by means of a tri-species hybrid. Turcotte and Feaster (1966) pointed out that two pairs of genes controlled anther color, and amphidiploid cottons that breed true for yellow pollen would have the genotype *P1P1P2P2*, while true-breeding cream-pollen strains would be *p1p1P2P2* or *p1p1p2p2*, and an orange-pollen mutant would have the genotype *P1P1p2p2*. Rhyne and Carter (1992) found a third locus, *P3*, and a true orange phenotype was conditioned by the genotype *P1P1p2p2p3p3*, but by then only the *P1* locus was associated with linkage groups (Chr.5; Percy and Kohel 1999). *Gossypium mustelinum* has yellow anthers and *G. hirsutum* has cream anthers. The segregation ratio for yellow: cream anther does not deviate significantly from the Mendelian ratio for a single gene (3:1) in the F_2_ population. The gene was further mapped on Chr.5, as reported in some previous research (Endrizzi *et al.* 1985; Rong *et al.* 2004; Yu *et al.* 2007; Liu *et al.* 2012); in addition, the anther color gene shared a common linked marker (A1535b) with Rong *et al.* (2004). The yellow color of most plant pollen is due to the presence of flavonoid and carotenoid pigments (Stanley and Linskens 1974). Further fine-mapping work could be performed to locate the gene controlling cotton anther color with the help of genome sequence data released recently.

In allotetraploid *Gossypium* species, yellow petal is controlled by duplicate dominant genes *Y1* (At subgenome) and *Y2* (Dt subgenome). *Y2* had been shown to be on Chr.18 (Endrizzi and Ray 1991), but *Y*1 had not been mapped to chromosome (Endrizzi *et al.* 1985). Guo *et al.* (2006) proposed that the *Y*1 gene might be anchored to one of several possible chromosomes, including the short arm of Chr.5, the long arms of Chr.11, Chr.8, Chr.13, or possibly Chr.10. Rong *et al.* (2004) assigned petal color to LGA01 (Chr.13). In our research, petal color was also mapped on Chr.13, associated with the interval between BNL2652a and PAR274.

The five petals of *G. hirsutum* have an area of anthocyanin pigmentation at the base, called a petal spot, which is conditioned by a gene called *R2* (Percy and Kohel 1999). Cultivated Upland cotton lacks a petal spot, whereas such spots are not uncommon in primitive cottons or race stocks (Fryxell 1984). Spotless petal was once used as the “hall-mark” of a commercially and agriculturally valuable stock of Pima cotton. For strains possessing this character, the fact that spotless is recessive would make it easy to recognize first generation hybrids resulting from accidental cross-pollination with normal Pima, as they would have a well-developed spot (Kbarnsy 1924). Rong *et al.* (2004) located the petal spot gene to two regions on Chr.1 and Chr.7. In our research, it was associated with the interval between A1625 and pAR040 on Chr.7, which is consistent with previous reports (Endrizzi *et al.* 1985; Yu *et al.* 2007; Lacape *et al.* 2009). Mapping of the three morphological markers, namely anther color, petal color, and petal spot in our research helps reveal or confirm their positons in cotton genome, which will benefit further research such as exploring the related genes controlling these traits.

### Colinearity of the HM map with the tetraploid cotton genome

To study colinearity and genome variations, dotplots were performed between the HM genetic map and the *G. hirsutum* reference genome (Figure 3). The overall marker order on the HM map agreed well with the corresponding sequences on the 26 major scaffolds of the *G. hirsutum* genome (Figure 3). Discrepancies in the orders along linkage groups and scaffolds were located in a few regions, which may be interpreted either as errors in genome assembly or in the construction of the genetic linkage map, or may be indicative of some structural rearrangements between different cotton species.

### Inversions between HM and other maps and their phylogenetic context

Our HM map consisted mainly of SSR and RFLP markers, making it comparable with other published maps, and suitable for a wide range of investigations in structural, functional, and evolutionary genomics. In most cases, the arrangements of genetic loci along the chromosomes of the HM map were the same as in HB, HT, and HD maps. Some locus order differences were likely due to reversals of neighboring markers explicable by occasional missing data in either population; meanwhile, some significant structural rearrangements were also observed (Figure 2 and Figure S1). There is evidence in some genomic regions of inversions that differentiate among HM, HT, HB, and HD maps (Figure 2D). The tetraploid phylogeny proposed by Grover *et al.* (2015a) facilitates phylogenetic inference regarding the origins of inversions between HM and other maps. No inversions were found to distinguish HM, HT, HB, and HD on Chr.1, Chr.6, Chr.10, Chr.11 Chr.14, Chr.16, Chr.21, Chr.22, and Chr.24 (Figure 2 and Figure S1), indicating that these nine chromosomes of *G. mustelinum*, *G. tomentosum*, *G. barbadense*, and *G. darwinii* have experienced little or no structural change since their divergence from a common ancestor.

On Chr.12, Chr.13, and Chr.18, HB and HD share common DNA marker orders, but each is inverted with respect to HM and HT (Figure 2 and Figure S1). The most parsimonious interpretation of this is that inversion occurred in a *G. barbadense–G. darwinii* common ancestor after its divergence from the *G. ekmanianum*–*G. hirsutum*–*G. tomentosum* clade. Less simply explained is a region of Chr.5 with common arrangement between HT and HD, but with each being inverted relative to HM and HB (Figure 2 and Figure S1). If some artifactual reason for this is not found, then it seems to suggest that *G. darwinii* and *G. tomentosum* have undergone similar changes, despite belonging to different tetraploid cotton clades. These species are both island endemics, and typically reside near coastlines with similar environments (*G. darwinii* is endemic to the Galapagos Islands, Wendel and Percy 1990; *G. tomentosum* is endemic to the Hawaiian Islands, DeJoode and Wendel 1992). Close relationships between these two island-endemic species has also been revealed in previous research (Wang *et al.* 2015).

Several inversions appear to be lineage-specific, presumably occurring more recently than the divergence of the affected tetraploid species from its nearest relative. Inversions limited to HM were found on Chr.3, Chr.15 and Chr.17; to HD on Chr.2, Chr.25 and Chr.26; to HT on Chr. 8 and Chr.19; and to HB on Chr.4, Chr.7, Chr.8, Chr.9, Chr.20, and Chr.23 (Figure 2 and Figure S1).

### Application of the HM map in QTL mapping

The *G. hirsutum* by *G. mustelinum* map was applied in mapping of fiber elongation QTL in advanced-backcross populations derived from the same parents. A total of 14 QTL was detected, with good reproducibility. Eight of the 14 QTL were detected simultaneously across different families or environments; six of the eight QTL were also mapped in the BC_3_F_2_ generation (Wang *et al.* 2016); and the QTL *qELO-19-1* was detected in three generations, and also by joint analysis across environments. While *G. mustelinum* produces little fiber, *G. mustelinum* alleles for four (28.6%) of the 14 QTL increased fiber elongation (Table 3), indicating the potential benefit of introgressing them into Upland cotton.

The identification of four transgressive QTL from this wild species provided one example of the value of our HM map. The HM map reported here is an important tool to elucidate cotton genome structure, and it may also aid in future genome sequencing of *G. mustelinum*. In addition, it will be beneficial to QTL analysis and facilitate cotton breeding with molecular marker technology.

## Supplementary Material

Supplemental Material
