# Supplementary material for: A Genetic Map Between Gossypium hirsutum and the Brazilian Endemic G. mustelinum and Its Application to QTL Mapping
Source: G3 (Bethesda). 2016 Mar 31;6(6):1673–85. doi: 10.1534/g3.116.029116 (PMC4889663; doi:10.1534/g3.116.029116)
Supplement: Supplemental Material [file supp_6_6_1673__index.html]

A Genetic Map Between Gossypium hirsutum and the Brazilian Endemic G. mustelinum and Its Application to QTL Mapping — Supplemental Material 

# A Genetic Map Between *Gossypium hirsutum* and the Brazilian Endemic *G. mustelinum* and Its Application to QTL Mapping

## Supplemental Material for Wang *et al.*, 2016

**Files in this Data Supplement:**

- Figure S1 - Comparison between *Gossypium hirsutum* × *G. mustelinum* (HM) genetic map with maps of *G. hirsutum* × *G. tomentosum* (HT), *G. hirsutum* × *G. barbadense* (HB), and *G. hirsutum* × *G. darwinii* (HD). (.pdf, 7,237 KB)
- File S1 - Marker data of *G. mustelinum* F2 mapping population. (.xls, 720 KB)
- File S2 - Data for QTL mapping. (.xlsx, 462 KB)
