# Supplementary material for: A Genetic Map Between Gossypium hirsutum and the Brazilian Endemic G. mustelinum and Its Application to QTL Mapping
Source: G3 (Bethesda). 2016 Mar 31;6(6):1673–85. doi: 10.1534/g3.116.029116 (PMC4889663; doi:10.1534/g3.116.029116)
Supplement: Supplemental Material [file supp_g3.116.029116_FigureS1.pdf]

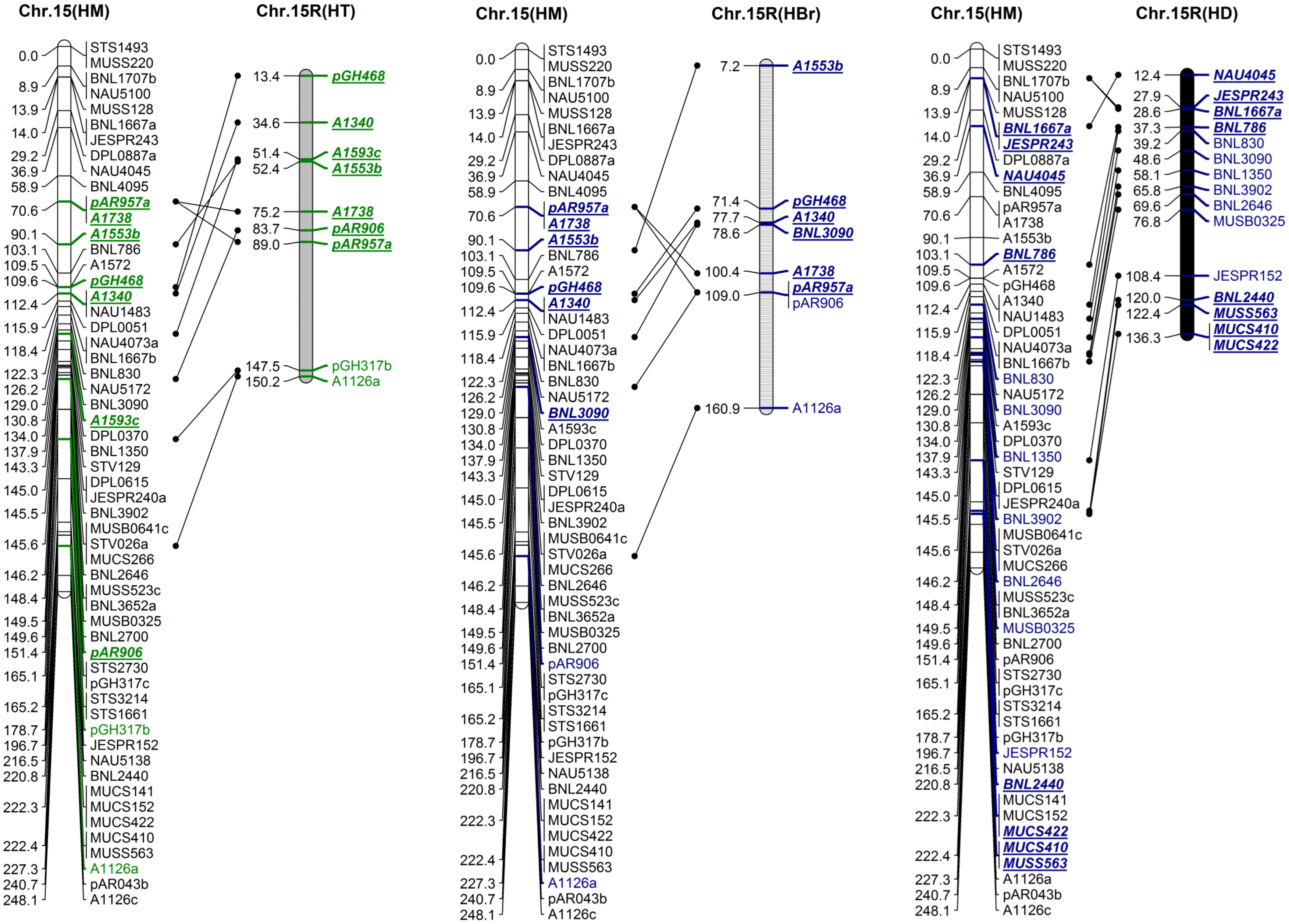
Chr.15(HM)
0.0 STS1493
MUSS220
8.9 BNL1707b
NAU5100
13.9 MUSS128
14.0 BNL1667a
JESPR243
29.2 DPL0887a
36.9 NAU4045
58.9 BNL4095
70.6 pAR957a
A1738
90.1 A1553b
103.1 BNL786
109.5 A1572
109.6 pGH468
A1340
112.4 NAU1483
115.9 DPL0051
NAU4073a
118.4 BNL1667b
122.3 BNL830
126.2 NAU5172
129.0 BNL3090
130.8 A1593c
134.0 DPL0370
137.9 BNL1350
143.3 STV129
DPL0615
145.0 JESPR240a
145.5 BNL3902
MUSB0641c
145.6 STV026a
MUCS266
146.2 BNL2646
148.4 MUSS523c
BNL3652a
149.5 MUSB0325
149.6 BNL2700
151.4 pAR906
165.1 STS2730
pGH317c
165.2 STS3214
STS1661
178.7 pGH317b
196.7 JESPR152
216.5 NAU5138
220.8 BNL2440
MUCS141
222.3 MUCS152
MUCS422
222.4 MUCS410
MUSS563
227.3 A1126a
240.7 pAR043b
248.1 A1126c
Chr.15R(HT)
13.4 pGH468
34.6 A1340
51.4 A1593c
52.4 A1553b
75.2 A1738
83.7 pAR906
89.0 pAR957a
147.5 pGH317b
150.2 A1126a
Chr.15R(HBr)
7.2 A1553b
71.4 pGH468
77.7 A1340
78.6 BNL3090
100.4 A1738
109.0 pAR957a
pAR906
160.9 A1126a
Chr.15R(HD)
12.4 NAU4045
27.9 JESPR243
28.6 BNL1667a
37.3 BNL786
39.2 BNL830
48.6 BNL3090
58.1 BNL1350
65.8 BNL3902
69.6 BNL2646
76.8 MUSB0325
108.4 JESPR152
120.0 BNL2440
122.4 MUSS563
136.3 MUCS410
MUCS422

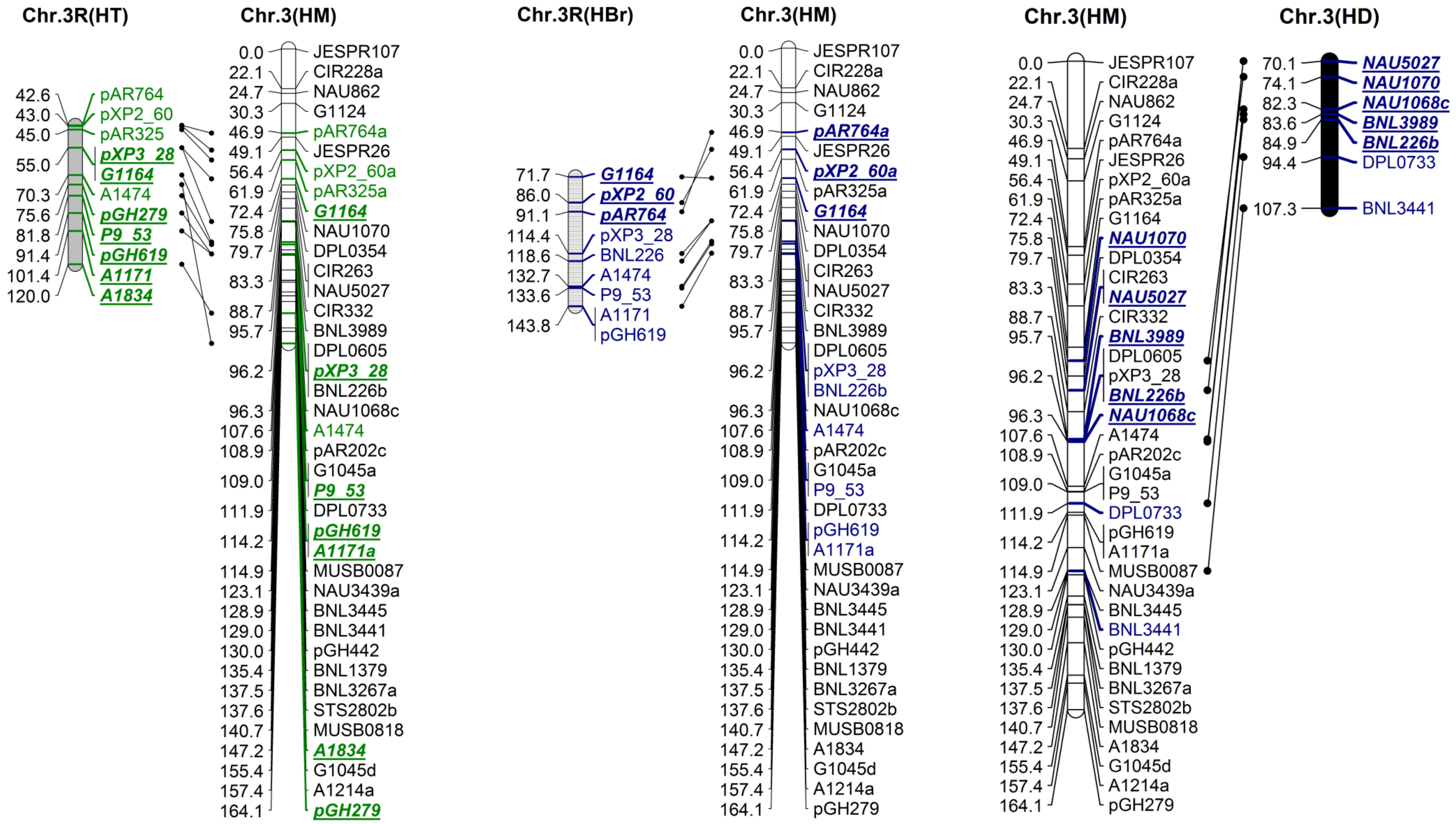
Chr.3R(HT)
42.6 43.0 45.0 55.0 70.3 75.6 81.8 91.4 101.4 120.0
pAR764 pXP2_60 pAR325 pXP3_28 G1164 A1474 pGH279 P9_53 pGH619 A1171 A1834
Chr.3(HM)
0.0 22.1 24.7 30.3 46.9 49.1 56.4 61.9 72.4 75.8 79.7 83.3 88.7 95.7 96.2 96.3 107.6 108.9 109.0 111.9 114.2 114.9 123.1 128.9 129.0 130.0 135.4 137.5 137.6 140.7 147.2 155.4 157.4 164.1
JESPR107 CIR228a NAU862 G1124 pAR764a JESPR26 pXP2_60a pAR325a G1164 NAU1070 DPL0354 CIR263 NAU5027 CIR332 BNL3989 DPL0605 pXP3_28 BNL226b NAU1068c A1474 pAR202c G1045a P9_53 DPL0733 pGH619 A1171a MUSB0087 NAU3439a BNL3445 BNL3441 pGH442 BNL1379 BNL3267a STS2802b MUSB0818 A1834 G1045d A1214a pGH279
Chr.3R(HBr)
71.7 86.0 91.1 114.4 118.6 132.7 133.6 143.8
G1164 pXP2_60 pAR764 pXP3_28 BNL226 A1474 P9_53 A1171 pGH619
Chr.3(HM)
0.0 22.1 24.7 30.3 46.9 49.1 56.4 61.9 72.4 75.8 79.7 83.3 88.7 95.7 96.2 96.3 107.6 108.9 109.0 111.9 114.2 114.9 123.1 128.9 129.0 130.0 135.4 137.5 137.6 140.7 147.2 155.4 157.4 164.1
JESPR107 CIR228a NAU862 G1124 pAR764a JESPR26 pXP2_60a pAR325a G1164 NAU1070 DPL0354 CIR263 NAU5027 CIR332 BNL3989 DPL0605 pXP3_28 BNL226b NAU1068c A1474 pAR202c G1045a P9_53 DPL0733 pGH619 A1171a MUSB0087 NAU3439a BNL3445 BNL3441 pGH442 BNL1379 BNL3267a STS2802b MUSB0818 A1834 G1045d A1214a pGH279
Chr.3(HM)
0.0 22.1 24.7 30.3 46.9 49.1 56.4 61.9 72.4 75.8 79.7 83.3 88.7 95.7 96.2 96.3 107.6 108.9 109.0 111.9 114.2 114.9 123.1 128.9 129.0 130.0 135.4 137.5 137.6 140.7 147.2 155.4 157.4 164.1
JESPR107 CIR228a NAU862 G1124 pAR764a JESPR26 pXP2_60a pAR325a G1164 NAU1070 DPL0354 CIR263 NAU5027 CIR332 BNL3989 DPL0605 pXP3_28 BNL226b NAU1068c A1474 pAR202c G1045a P9_53 DPL0733 pGH619 A1171a MUSB0087 NAU3439a BNL3445 BNL3441 pGH442 BNL1379 BNL3267a STS2802b MUSB0818 A1834 G1045d A1214a pGH279
Chr.3(HD)
70.1 74.1 82.3 83.6 84.9 94.4 107.3
NAU5027 NAU1070 NAU1068c BNL3989 BNL226b DPL0733 BNL3441

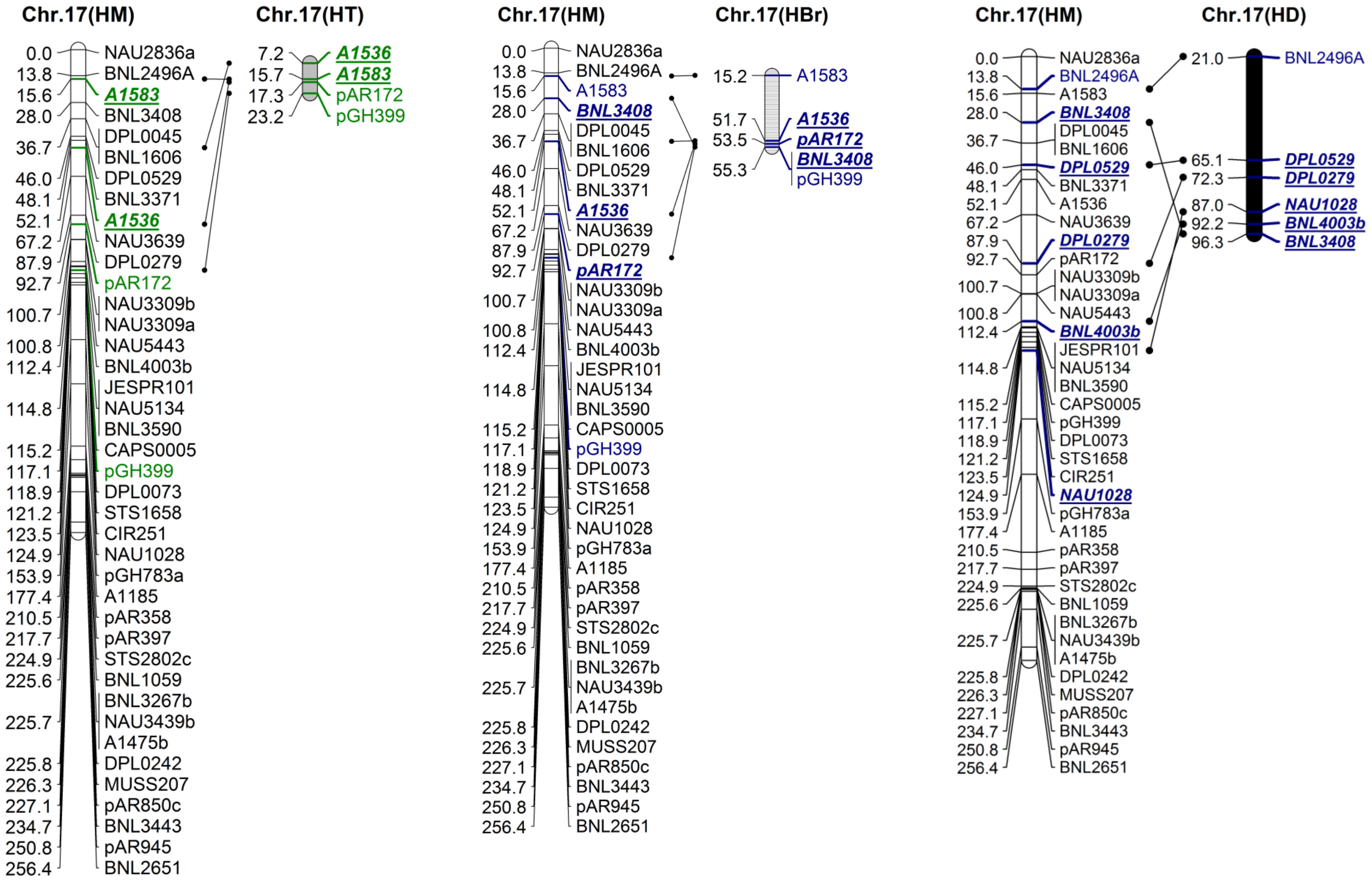

Chr.17(HM)
Chr.17(HT)
Chr.17(HM)
Chr.17(HBr)
Chr.17(HM)
Chr.17(HD)
0.0 NAU2836a
13.8 BNL2496A
15.6 A1583
28.0 BNL3408
36.7 DPL0045
BNL1606
46.0 DPL0529
48.1 BNL3371
52.1 A1536
67.2 NAU3639
87.9 DPL0279
92.7 pAR172
100.7 NAU3309b
NAU3309a
100.8 NAU5443
112.4 BNL4003b
JESPR101
114.8 NAU5134
BNL3590
115.2 CAPS0005
117.1 pGH399
118.9 DPL0073
121.2 STS1658
123.5 CIR251
124.9 NAU1028
153.9 pGH783a
177.4 A1185
210.5 pAR358
217.7 pAR397
224.9 STS2802c
225.6 BNL1059
BNL3267b
225.7 NAU3439b
A1475b
225.8 DPL0242
226.3 MUSS207
227.1 pAR850c
234.7 BNL3443
250.8 pAR945
256.4 BNL2651
7.2 A1536
15.7 A1583
17.3 pAR172
23.2 pGH399
15.2 A1583
51.7 A1536
53.5 pAR172
55.3 BNL3408
pGH399
21.0 BNL2496A
65.1 DPL0529
72.3 DPL0279
87.0 NAU1028
92.2 BNL4003b
96.3 BNL3408

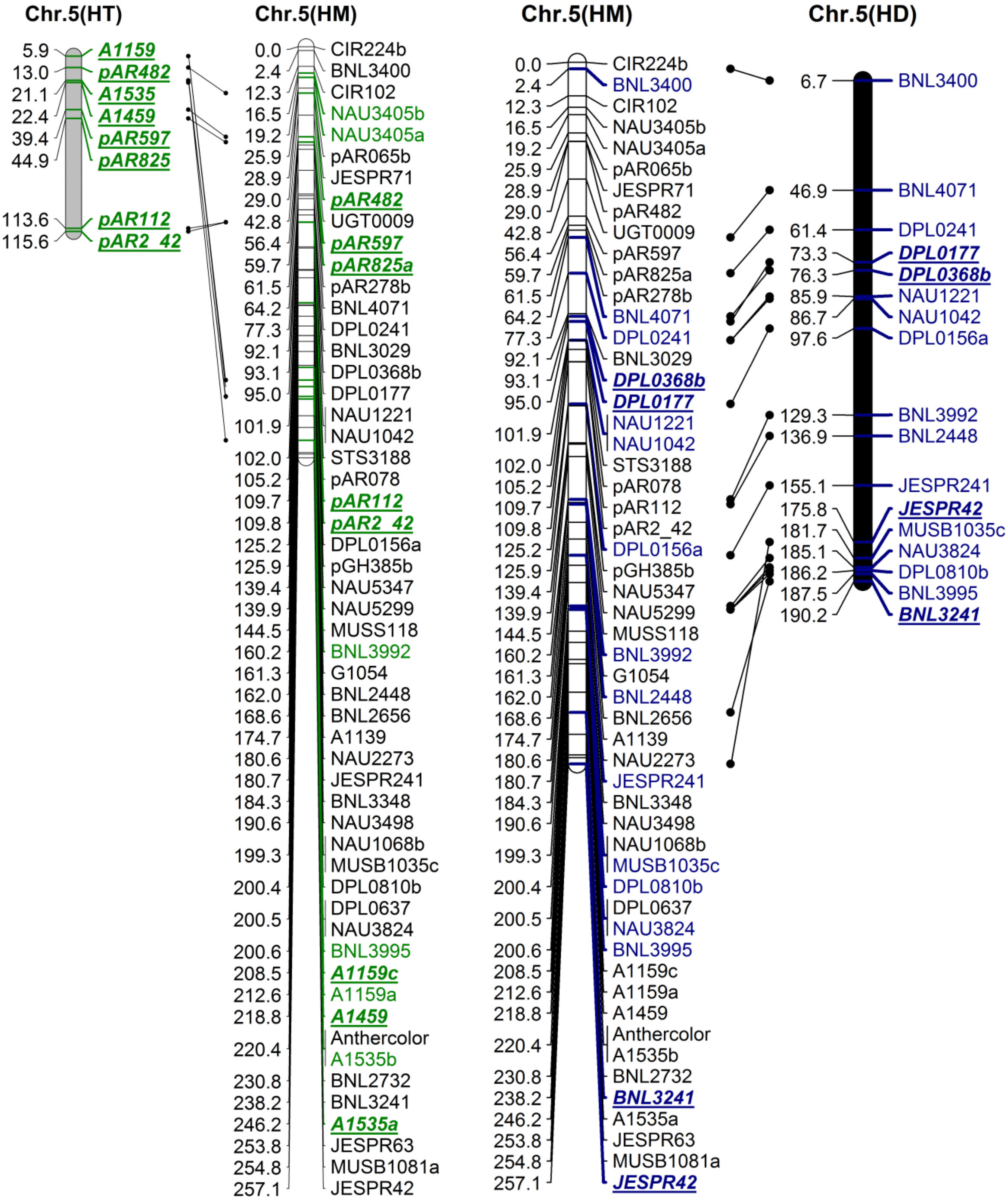

Chr.5(HT)
Chr.5(HM)
Chr.5(HM)
Chr.5(HD)
A1159
pAR482
A1535
A1459
pAR597
pAR825
pAR112
pAR2_42
CIR224b
BNL3400
CIR102
NAU3405b
NAU3405a
pAR065b
JESPR71
pAR482
UGT0009
pAR597
pAR825a
pAR278b
BNL4071
DPL0241
BNL3029
DPL0368b
DPL0177
NAU1221
NAU1042
STS3188
pAR078
pAR112
pAR2_42
DPL0156a
pGH385b
NAU5347
NAU5299
MUSS118
BNL3992
G1054
BNL2448
BNL2656
A1139
NAU2273
JESPR241
BNL3348
NAU3498
NAU1068b
MUSB1035c
DPL0810b
DPL0637
NAU3824
BNL3995
A1159c
A1159a
A1459
Anthercolor
A1535b
BNL2732
BNL3241
A1535a
JESPR63
MUSB1081a
JESPR42

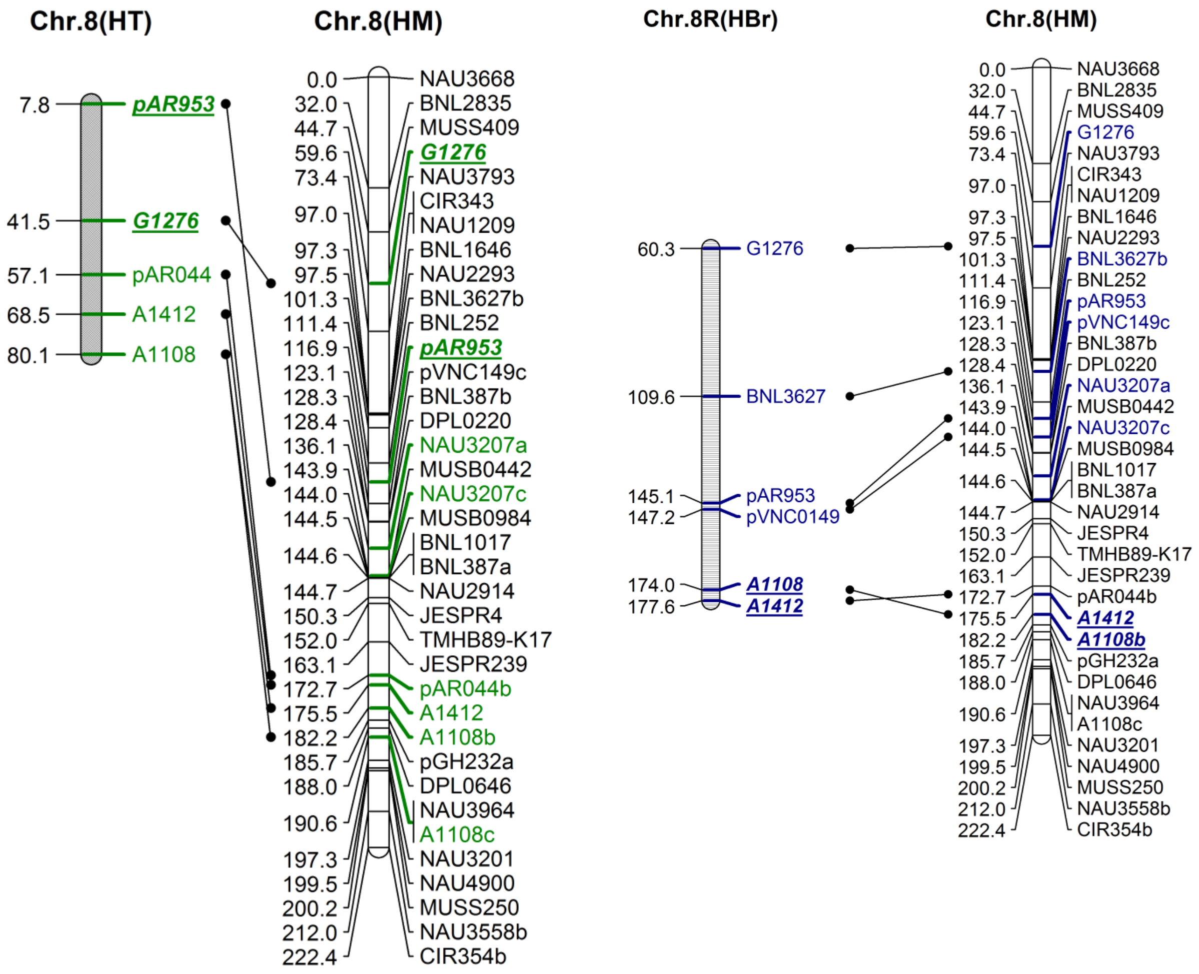

Chr.8(HT)
7.8 pAR953
41.5 G1276
57.1 pAR044
68.5 A1412
80.1 A1108
Chr.8(HM)
0.0 NAU3668
32.0 BNL2835
44.7 MUSS409
59.6 G1276
73.4 NAU3793
97.0 CIR343
NAU1209
97.3 BNL1646
97.5 NAU2293
101.3 BNL3627b
111.4 BNL252
116.9 pAR953
123.1 pVNC149c
128.3 BNL387b
128.4 DPL0220
136.1 NAU3207a
143.9 MUSB0442
144.0 NAU3207c
144.5 MUSB0984
144.6 BNL1017
BNL387a
144.7 NAU2914
150.3 JESPR4
152.0 TMHB89-K17
163.1 JESPR239
172.7 pAR044b
175.5 A1412
182.2 A1108b
185.7 pGH232a
188.0 DPL0646
190.6 NAU3964
A1108c
197.3 NAU3201
199.5 NAU4900
200.2 MUSS250
212.0 NAU3558b
222.4 CIR354b
Chr.8R(HBr)
60.3 G1276
109.6 BNL3627
145.1 pAR953
147.2 pVNC0149
174.0 A1108
177.6 A1412
Chr.8(HM)
0.0 NAU3668
32.0 BNL2835
44.7 MUSS409
59.6 G1276
73.4 NAU3793
97.0 CIR343
NAU1209
97.3 BNL1646
97.5 NAU2293
101.3 BNL3627b
111.4 BNL252
116.9 pAR953
123.1 pVNC149c
128.3 BNL387b
128.4 DPL0220
136.1 NAU3207a
143.9 MUSB0442
144.0 NAU3207c
144.5 MUSB0984
144.6 BNL1017
BNL387a
144.7 NAU2914
150.3 JESPR4
152.0 TMHB89-K17
163.1 JESPR239
172.7 pAR044b
175.5 A1412
182.2 A1108b
185.7 pGH232a
188.0 DPL0646
190.6 NAU3964
A1108c
197.3 NAU3201
199.5 NAU4900
200.2 MUSS250
212.0 NAU3558b
222.4 CIR354b

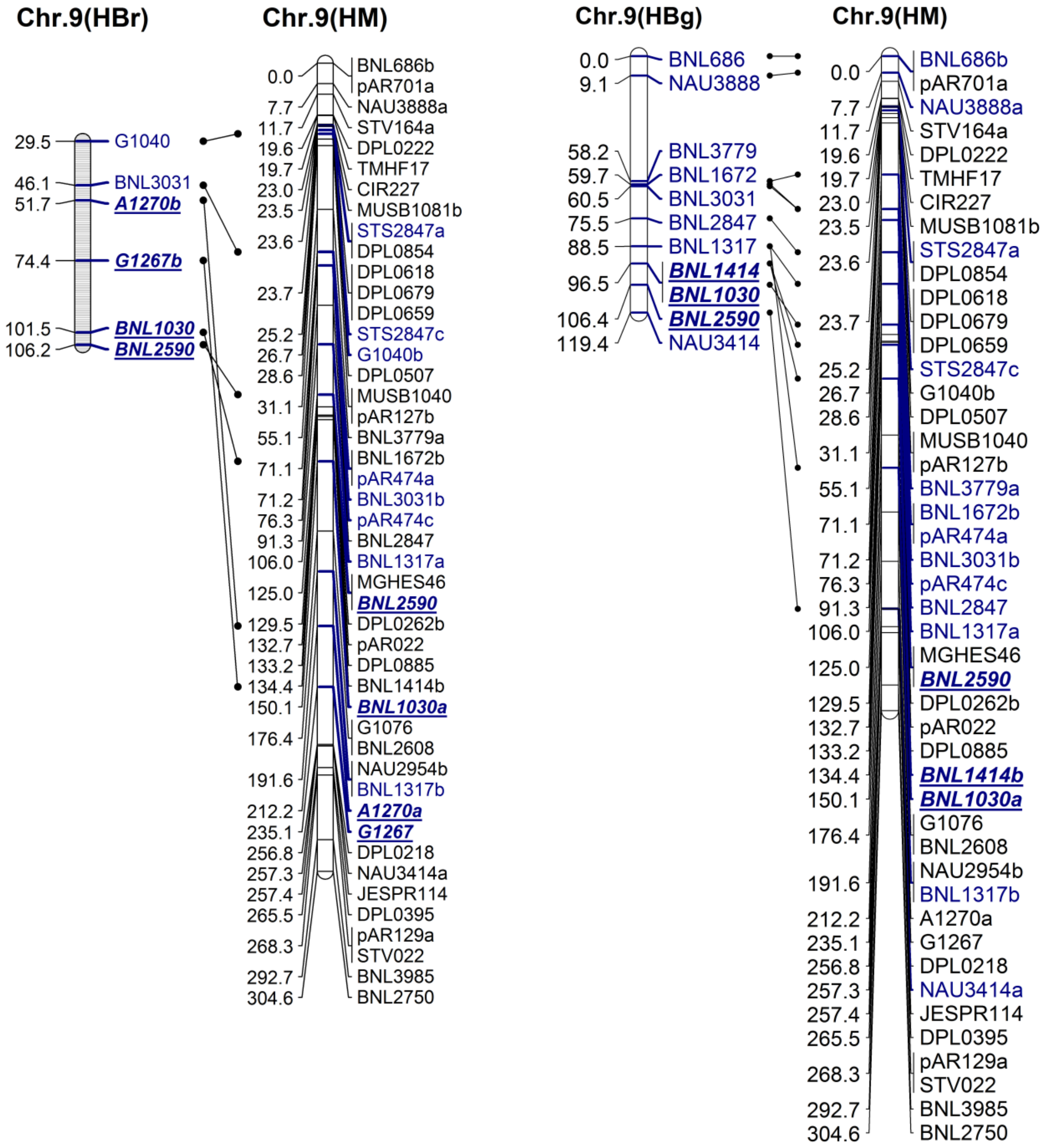

Chr.9(HBr)
29.5 G1040
46.1 BNL3031
51.7 A1270b
74.4 G1267b
101.5 BNL1030
106.2 BNL2590
Chr.9(HM)
0.0
7.7
11.7
19.6
19.7
23.0
23.5
23.6
23.7
25.2
26.7
28.6
31.1
55.1
71.1
71.2
76.3
91.3
106.0
125.0
129.5
132.7
133.2
134.4
150.1
176.4
191.6
212.2
235.1
256.8
257.3
257.4
265.5
268.3
292.7
304.6
BNL686b
pAR701a
NAU3888a
STV164a
DPL0222
TMHF17
CIR227
MUSB1081b
STS2847a
DPL0854
DPL0618
DPL0679
DPL0659
STS2847c
G1040b
DPL0507
MUSB1040
pAR127b
BNL3779a
BNL1672b
pAR474a
BNL3031b
pAR474c
BNL2847
BNL1317a
MGHES46
BNL2590
DPL0262b
pAR022
DPL0885
BNL1414b
BNL1030a
G1076
BNL2608
NAU2954b
BNL1317b
A1270a
G1267
DPL0218
NAU3414a
JESPR114
DPL0395
pAR129a
STV022
BNL3985
BNL2750
Chr.9(HBg)
0.0 BNL686
9.1 NAU3888
58.2 BNL3779
59.7 BNL1672
60.5 BNL3031
75.5 BNL2847
88.5 BNL1317
96.5 BNL1414
BNL1030
106.4 BNL2590
119.4 NAU3414
Chr.9(HM)
0.0
7.7
11.7
19.6
19.7
23.0
23.5
23.6
23.7
25.2
26.7
28.6
31.1
55.1
71.1
71.2
76.3
91.3
106.0
125.0
129.5
132.7
133.2
134.4
150.1
176.4
191.6
212.2
235.1
256.8
257.3
257.4
265.5
268.3
292.7
304.6
BNL686b
pAR701a
NAU3888a
STV164a
DPL0222
TMHF17
CIR227
MUSB1081b
STS2847a
DPL0854
DPL0618
DPL0679
DPL0659
STS2847c
G1040b
DPL0507
MUSB1040
pAR127b
BNL3779a
BNL1672b
pAR474a
BNL3031b
pAR474c
BNL2847
BNL1317a
MGHES46
BNL2590
DPL0262b
pAR022
DPL0885
BNL1414b
BNL1030a
G1076
BNL2608
NAU2954b
BNL1317b
A1270a
G1267
DPL0218
NAU3414a
JESPR114
DPL0395
pAR129a
STV022
BNL3985
BNL2750

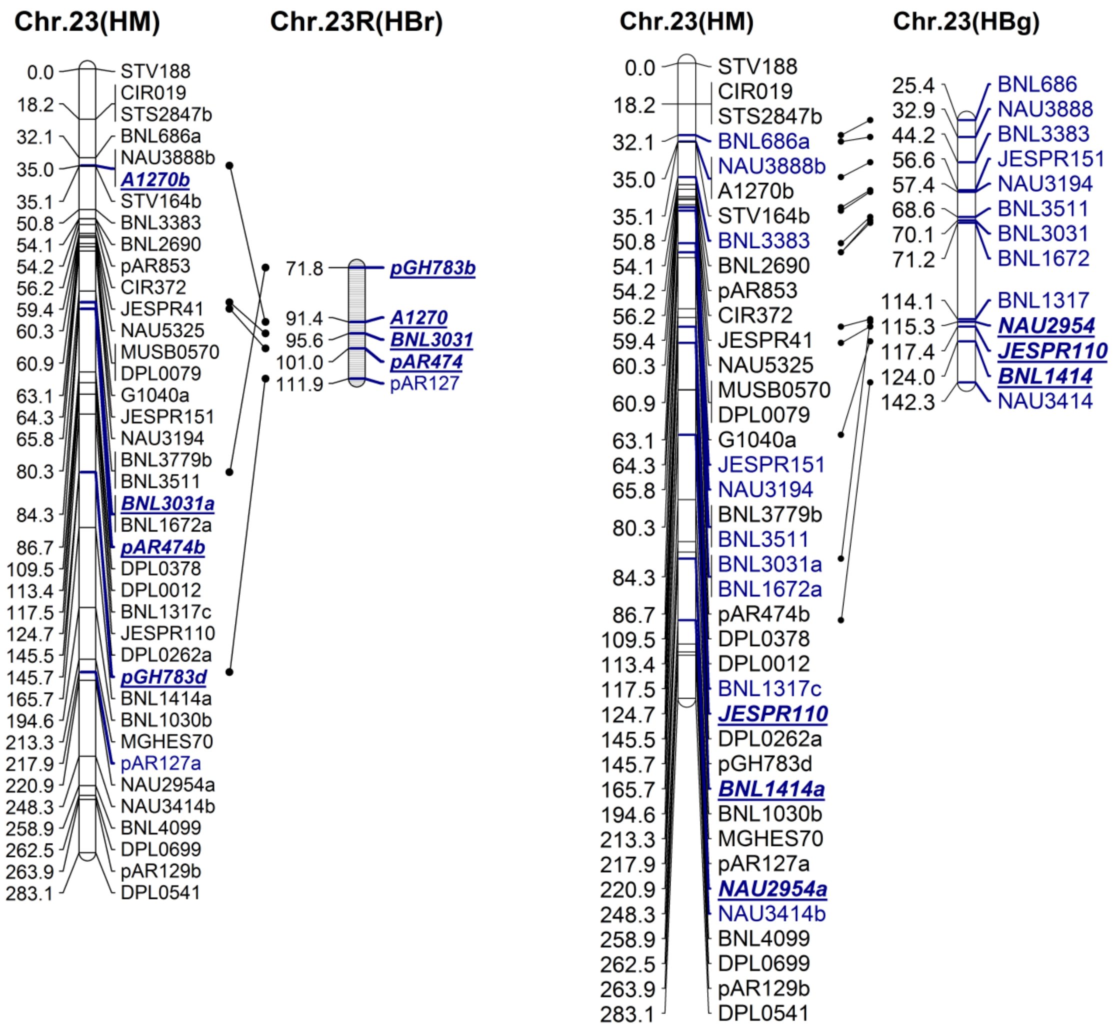

Chr.23(HM)
Chr.23R(HBr)
Chr.23(HM)
Chr.23(HBg)
0.0
18.2
32.1
35.0
35.1
50.8
54.1
54.2
56.2
59.4
60.3
60.9
63.1
64.3
65.8
80.3
84.3
86.7
109.5
113.4
117.5
124.7
145.5
145.7
165.7
194.6
213.3
217.9
220.9
248.3
258.9
262.5
263.9
283.1
STV188
CIR019
STS2847b
BNL686a
NAU3888b
A1270b
STV164b
BNL3383
BNL2690
pAR853
CIR372
JESPR41
NAU5325
MUSB0570
DPL0079
G1040a
JESPR151
NAU3194
BNL3779b
BNL3511
BNL3031a
BNL1672a
pAR474b
DPL0378
DPL0012
BNL1317c
JESPR110
DPL0262a
pGH783d
BNL1414a
BNL1030b
MGHES70
pAR127a
NAU2954a
NAU3414b
BNL4099
DPL0699
pAR129b
DPL0541
71.8
91.4
95.6
101.0
111.9
pGH783b
A1270
BNL3031
pAR474
pAR127
25.4
32.9
44.2
56.6
57.4
68.6
70.1
71.2
114.1
115.3
117.4
124.0
142.3
BNL686
NAU3888
BNL3383
JESPR151
NAU3194
BNL3511
BNL3031
BNL1672
BNL1317
NAU2954
JESPR110
BNL1414
NAU3414

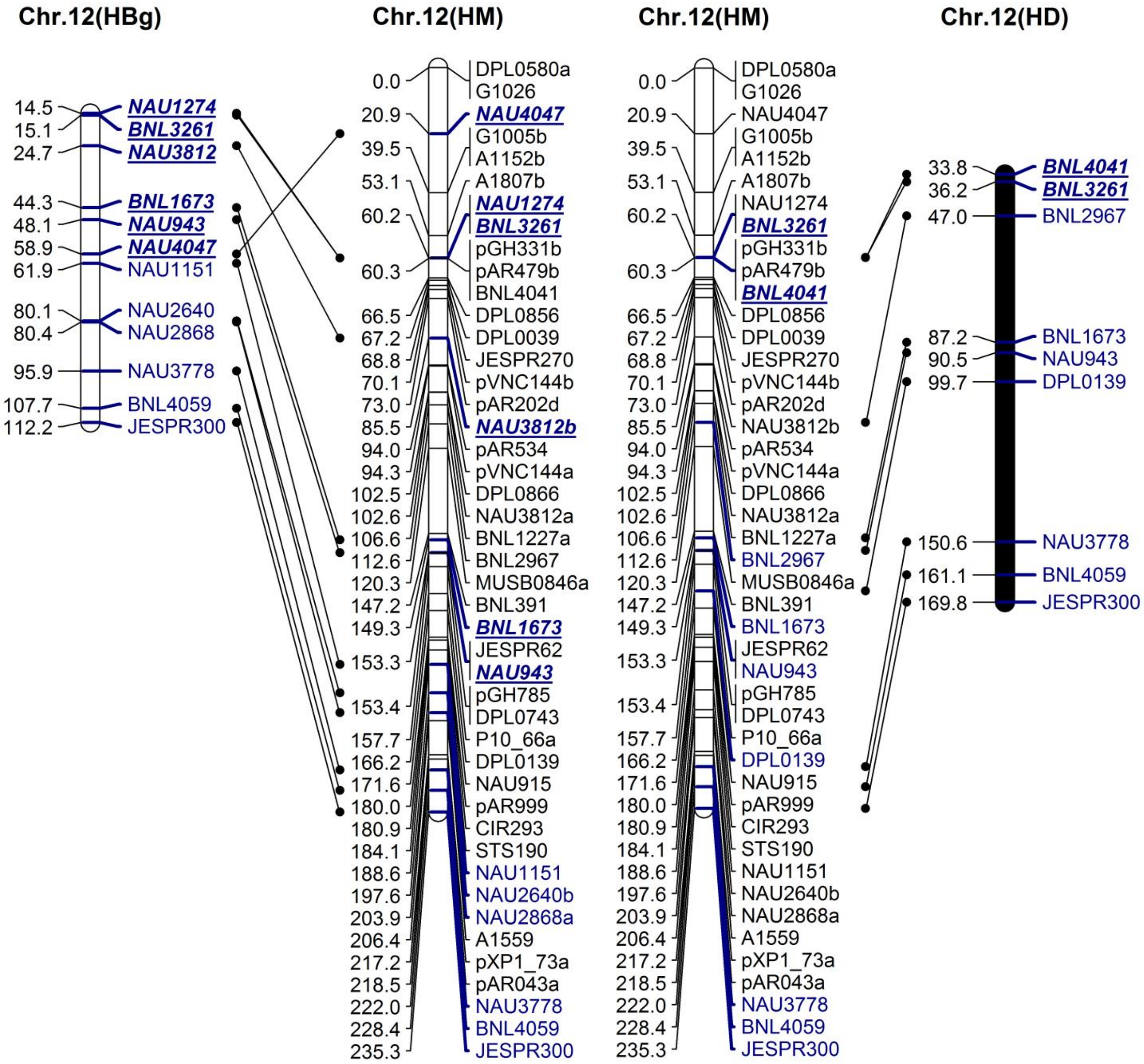

Chr.12(HBg)
14.5 NAU1274
15.1 BNL3261
24.7 NAU3812
44.3 BNL1673
48.1 NAU943
58.9 NAU4047
61.9 NAU1151
80.1 NAU2640
80.4 NAU2868
95.9 NAU3778
107.7 BNL4059
112.2 JESPR300
Chr.12(HM)
0.0
20.9
39.5
53.1
60.2
60.3
66.5
67.2
68.8
70.1
73.0
85.5
94.0
94.3
102.5
102.6
106.6
112.6
120.3
147.2
149.3
153.3
153.4
157.7
166.2
171.6
180.0
180.9
184.1
188.6
197.6
203.9
206.4
217.2
218.5
222.0
228.4
235.3
DPL0580a
G1026
NAU4047
G1005b
A1152b
A1807b
NAU1274
BNL3261
pGH331b
pAR479b
BNL4041
DPL0856
DPL0039
JESPR270
pVNC144b
pAR202d
NAU3812b
pAR534
pVNC144a
DPL0866
NAU3812a
BNL1227a
BNL2967
MUSB0846a
BNL391
BNL1673
JESPR62
NAU943
pGH785
DPL0743
P10_66a
DPL0139
NAU915
pAR999
CIR293
STS190
NAU1151
NAU2640b
NAU2868a
A1559
pXP1_73a
pAR043a
NAU3778
BNL4059
JESPR300
Chr.12(HM)
0.0
20.9
39.5
53.1
60.2
60.3
66.5
67.2
68.8
70.1
73.0
85.5
94.0
94.3
102.5
102.6
106.6
112.6
120.3
147.2
149.3
153.3
153.4
157.7
166.2
171.6
180.0
180.9
184.1
188.6
197.6
203.9
206.4
217.2
218.5
222.0
228.4
235.3
DPL0580a
G1026
NAU4047
G1005b
A1152b
A1807b
NAU1274
BNL3261
pGH331b
pAR479b
BNL4041
DPL0856
DPL0039
JESPR270
pVNC144b
pAR202d
NAU3812b
pAR534
pVNC144a
DPL0866
NAU3812a
BNL1227a
BNL2967
MUSB0846a
BNL391
BNL1673
JESPR62
NAU943
pGH785
DPL0743
P10_66a
DPL0139
NAU915
pAR999
CIR293
STS190
NAU1151
NAU2640b
NAU2868a
A1559
pXP1_73a
pAR043a
NAU3778
BNL4059
JESPR300
Chr.12(HD)
33.8 BNL4041
36.2 BNL3261
47.0 BNL2967
87.2 BNL1673
90.5 NAU943
99.7 DPL0139
150.6 NAU3778
161.1 BNL4059
169.8 JESPR300

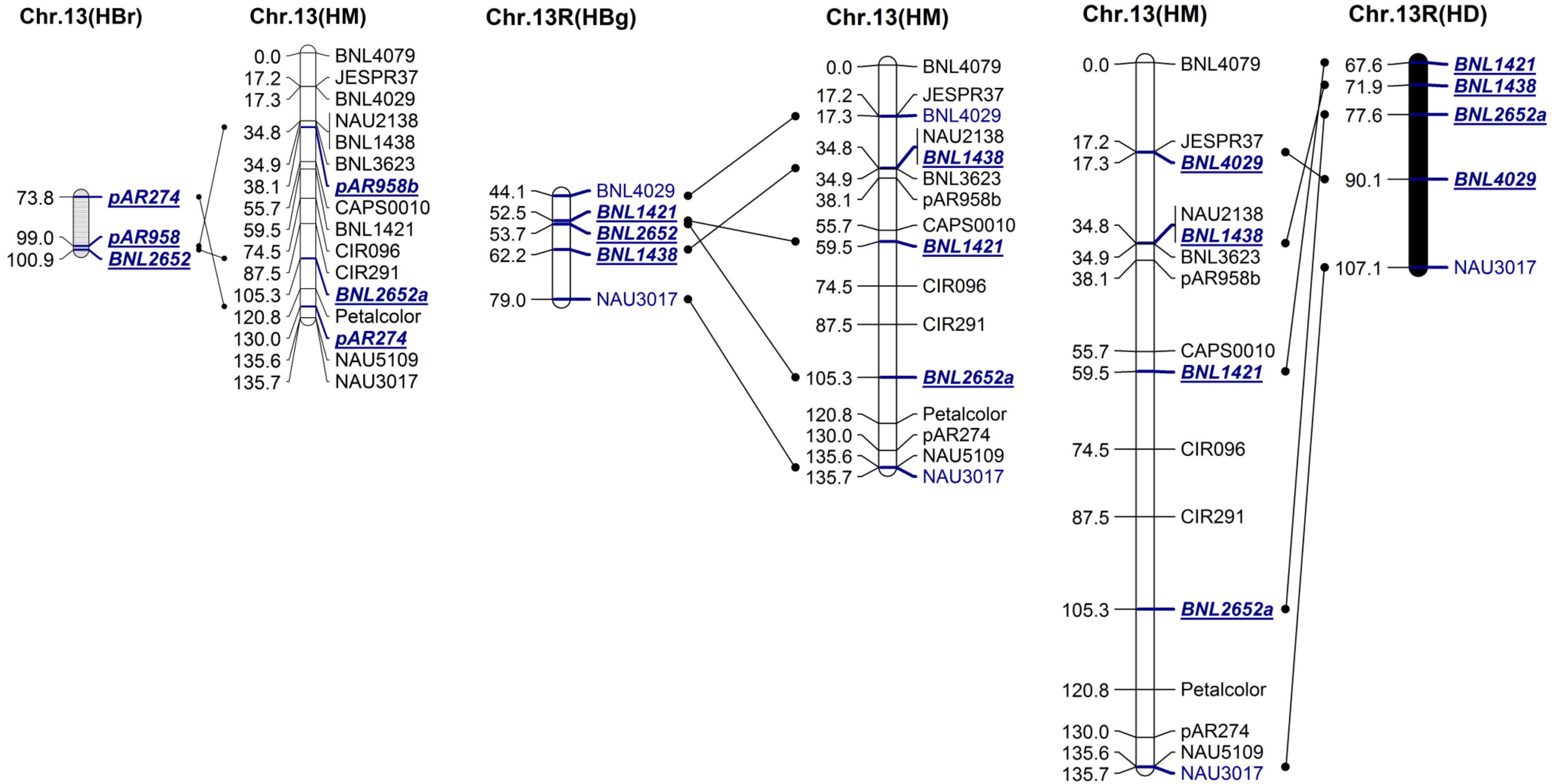

Chr.13(HBr)
73.8 pAR274
99.0 pAR958
100.9 BNL2652
Chr.13(HM)
0.0 BNL4079
17.2 JESPR37
17.3 BNL4029
34.8 NAU2138
BNL1438
34.9 BNL3623
38.1 pAR958b
55.7 CAPS0010
59.5 BNL1421
74.5 CIR096
87.5 CIR291
105.3 BNL2652a
120.8 Petalcolor
130.0 pAR274
135.6 NAU5109
135.7 NAU3017
Chr.13R(HBg)
44.1 BNL4029
52.5 BNL1421
53.7 BNL2652
62.2 BNL1438
79.0 NAU3017
Chr.13(HM)
0.0 BNL4079
17.2 JESPR37
17.3 BNL4029
34.8 NAU2138
BNL1438
34.9 BNL3623
38.1 pAR958b
55.7 CAPS0010
59.5 BNL1421
74.5 CIR096
87.5 CIR291
105.3 BNL2652a
120.8 Petalcolor
130.0 pAR274
135.6 NAU5109
135.7 NAU3017
Chr.13(HM)
0.0 BNL4079
17.2 JESPR37
17.3 BNL4029
34.8 NAU2138
BNL1438
34.9 BNL3623
38.1 pAR958b
55.7 CAPS0010
59.5 BNL1421
74.5 CIR096
87.5 CIR291
105.3 BNL2652a
120.8 Petalcolor
130.0 pAR274
135.6 NAU5109
135.7 NAU3017
Chr.13R(HD)
67.6 BNL1421
71.9 BNL1438
77.6 BNL2652a
90.1 BNL4029
107.1 NAU3017

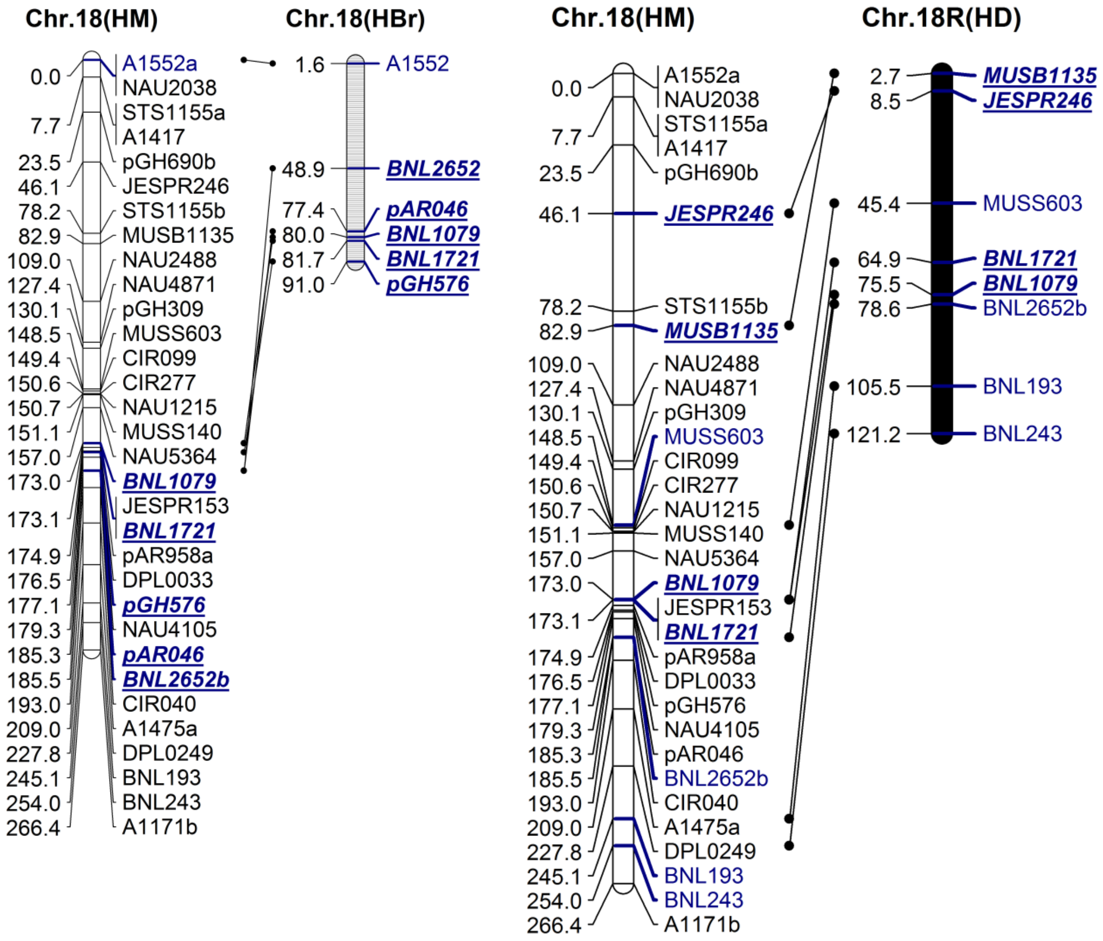

Chr.18(HM)
Chr.18(HBr)
Chr.18(HM)
Chr.18R(HD)
0.0 A1552a
NAU2038
7.7 STS1155a
A1417
23.5 pGH690b
46.1 JESPR246
78.2 STS1155b
82.9 MUSB1135
109.0 NAU2488
127.4 NAU4871
130.1 pGH309
148.5 MUSS603
149.4 CIR099
150.6 CIR277
150.7 NAU1215
151.1 MUSS140
157.0 NAU5364
173.0 BNL1079
JESPR153
173.1 BNL1721
174.9 pAR958a
176.5 DPL0033
177.1 pGH576
179.3 NAU4105
185.3 pAR046
185.5 BNL2652b
193.0 CIR040
209.0 A1475a
227.8 DPL0249
245.1 BNL193
254.0 BNL243
266.4 A1171b
1.6 A1552
48.9 BNL2652
77.4 pAR046
80.0 BNL1079
81.7 BNL1721
91.0 pGH576
2.7 MUSB1135
8.5 JESPR246
45.4 MUSS603
64.9 BNL1721
75.5 BNL1079
78.6 BNL2652b
105.5 BNL193
121.2 BNL243

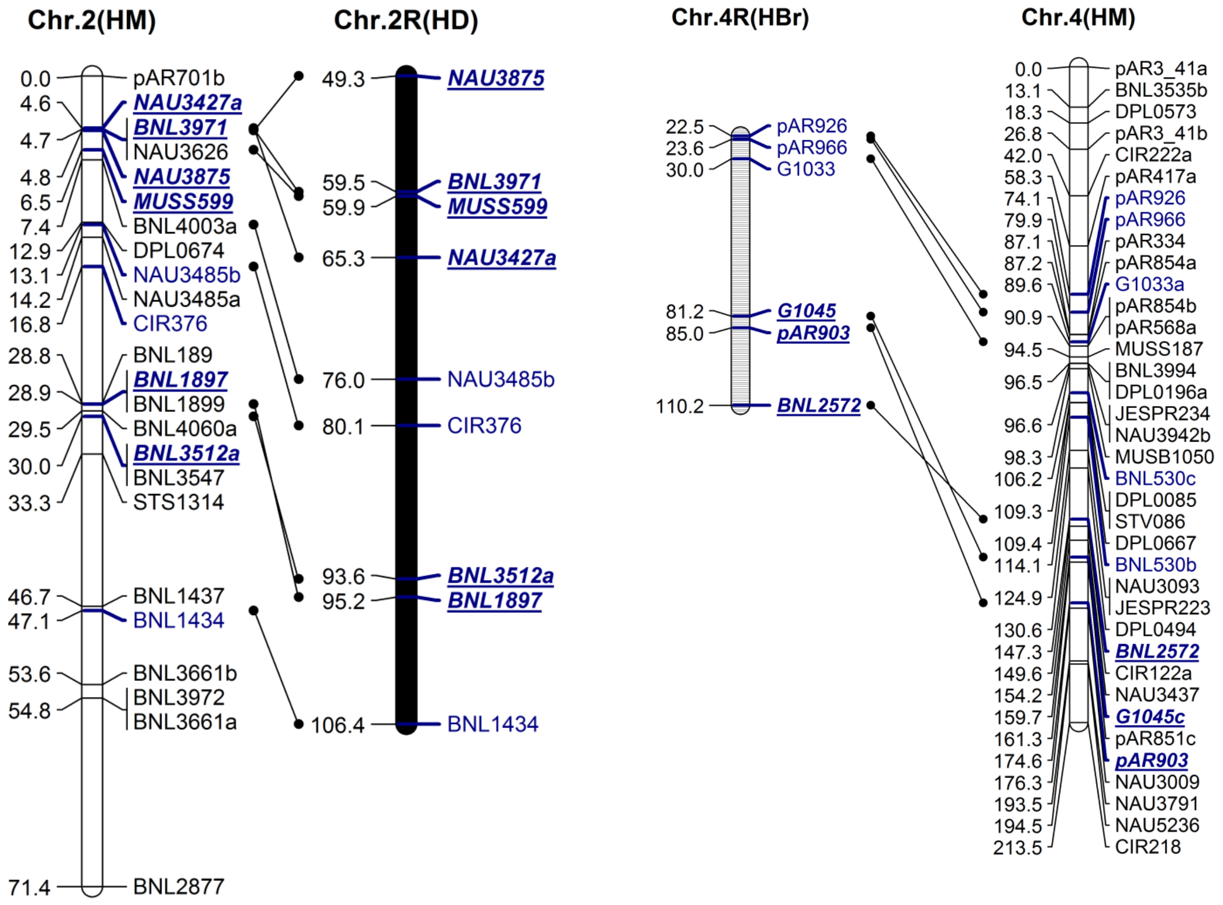
Chr.2(HM)
0.0 pAR701b
4.6 NAU3427a
4.7 BNL3971
NAU3626
4.8 NAU3875
6.5 MUSS599
7.4 BNL4003a
12.9 DPL0674
13.1 NAU3485b
14.2 NAU3485a
16.8 CIR376
28.8 BNL189
28.9 BNL1897
BNL1899
29.5 BNL4060a
30.0 BNL3512a
BNL3547
33.3 STS1314
46.7 BNL1437
47.1 BNL1434
53.6 BNL3661b
54.8 BNL3972
BNL3661a
71.4 BNL2877
Chr.2R(HD)
49.3 NAU3875
59.5 BNL3971
59.9 MUSS599
65.3 NAU3427a
76.0 NAU3485b
80.1 CIR376
93.6 BNL3512a
95.2 BNL1897
106.4 BNL1434
Chr.4R(HBr)
22.5 pAR926
23.6 pAR966
30.0 G1033
81.2 G1045
85.0 pAR903
110.2 BNL2572
Chr.4(HM)
0.0 pAR3_41a
13.1 BNL3535b
18.3 DPL0573
26.8 pAR3_41b
42.0 CIR222a
58.3 pAR417a
74.1 pAR926
79.9 pAR966
87.1 pAR334
87.2 pAR854a
89.6 G1033a
90.9 pAR854b
pAR568a
94.5 MUSS187
96.5 BNL3994
DPL0196a
96.6 JESPR234
NAU3942b
98.3 MUSB1050
106.2 BNL530c
109.3 DPL0085
STV086
109.4 DPL0667
114.1 BNL530b
124.9 NAU3093
JESPR223
130.6 DPL0494
147.3 BNL2572
149.6 CIR122a
154.2 NAU3437
159.7 G1045c
161.3 pAR851c
174.6 pAR903
176.3 NAU3009
193.5 NAU3791
194.5 NAU5236
213.5 CIR218

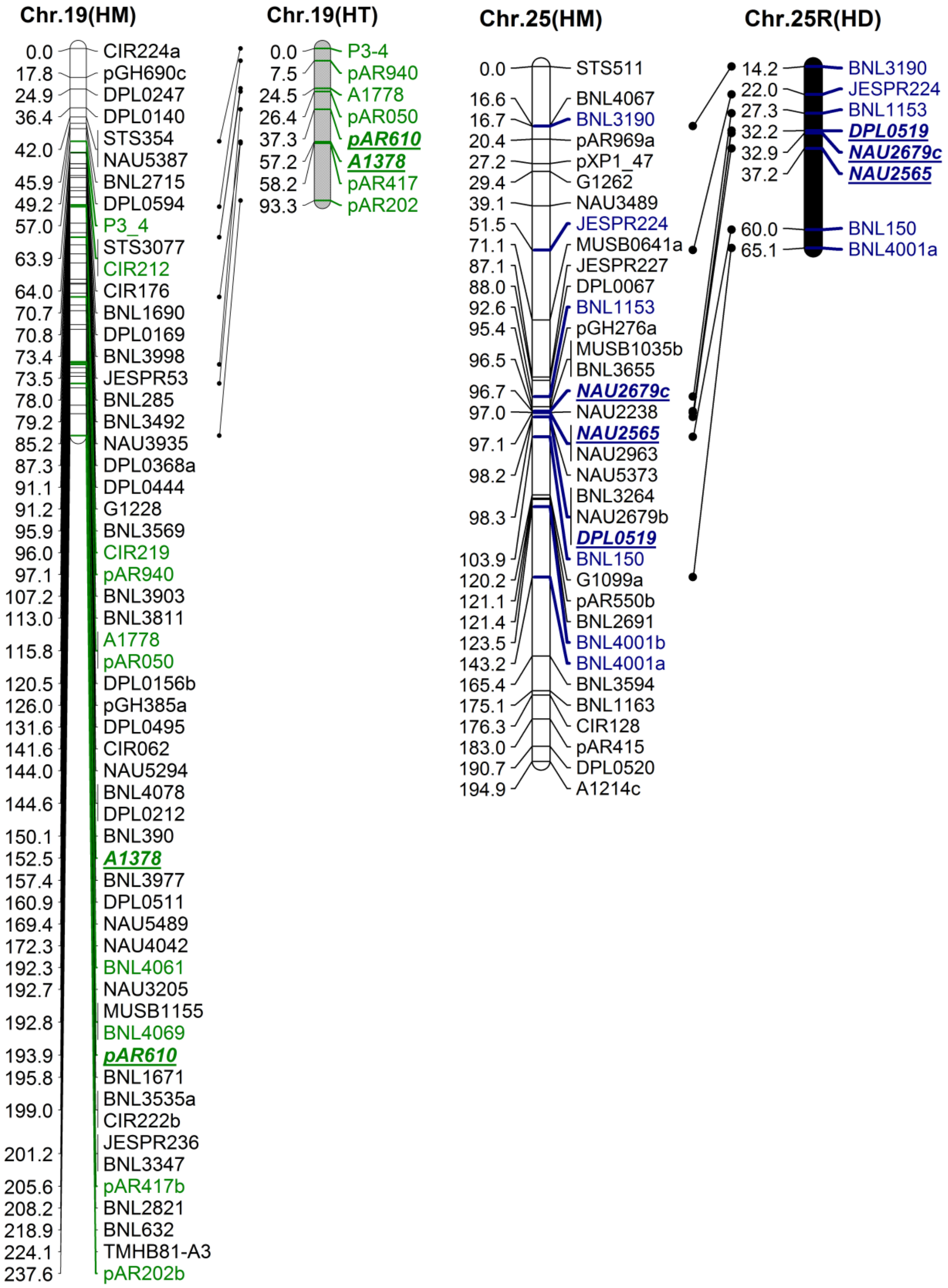

Chr.19(HM)
0.0 CIR224a
17.8 pGH690c
24.9 DPL0247
36.4 DPL0140
STS354
42.0 NAU5387
45.9 BNL2715
49.2 DPL0594
57.0 P3_4
STS3077
63.9 CIR212
64.0 CIR176
70.7 BNL1690
70.8 DPL0169
73.4 BNL3998
73.5 JESPR53
78.0 BNL285
79.2 BNL3492
85.2 NAU3935
87.3 DPL0368a
91.1 DPL0444
91.2 G1228
95.9 BNL3569
96.0 CIR219
97.1 pAR940
107.2 BNL3903
113.0 BNL3811
A1778
115.8 pAR050
120.5 DPL0156b
126.0 pGH385a
131.6 DPL0495
141.6 CIR062
144.0 NAU5294
BNL4078
144.6 DPL0212
150.1 BNL390
152.5 A1378
157.4 BNL3977
160.9 DPL0511
169.4 NAU5489
172.3 NAU4042
192.3 BNL4061
192.7 NAU3205
MUSB1155
192.8 BNL4069
193.9 pAR610
195.8 BNL1671
BNL3535a
199.0 CIR222b
JESPR236
201.2 BNL3347
205.6 pAR417b
208.2 BNL2821
218.9 BNL632
224.1 TMHB81-A3
237.6 pAR202b
Chr.19(HT)
0.0 P3-4
7.5 pAR940
24.5 A1778
26.4 pAR050
37.3 pAR610
57.2 A1378
58.2 pAR417
93.3 pAR202
Chr.25(HM)
0.0 STS511
16.6 BNL4067
16.7 BNL3190
20.4 pAR969a
27.2 pXP1_47
29.4 G1262
39.1 NAU3489
51.5 JESPR224
71.1 MUSB0641a
87.1 JESPR227
88.0 DPL0067
92.6 BNL1153
95.4 pGH276a
MUSB1035b
96.5 BNL3655
96.7 NAU2679c
97.0 NAU2238
NAU2565
97.1 NAU2963
98.2 NAU5373
BNL3264
98.3 NAU2679b
DPL0519
103.9 BNL150
120.2 G1099a
121.1 pAR550b
121.4 BNL2691
123.5 BNL4001b
143.2 BNL4001a
165.4 BNL3594
175.1 BNL1163
176.3 CIR128
183.0 pAR415
190.7 DPL0520
194.9 A1214c
Chr.25R(HD)
14.2 BNL3190
22.0 JESPR224
27.3 BNL1153
32.2 DPL0519
32.9 NAU2679c
37.2 NAU2565
60.0 BNL150
65.1 BNL4001a

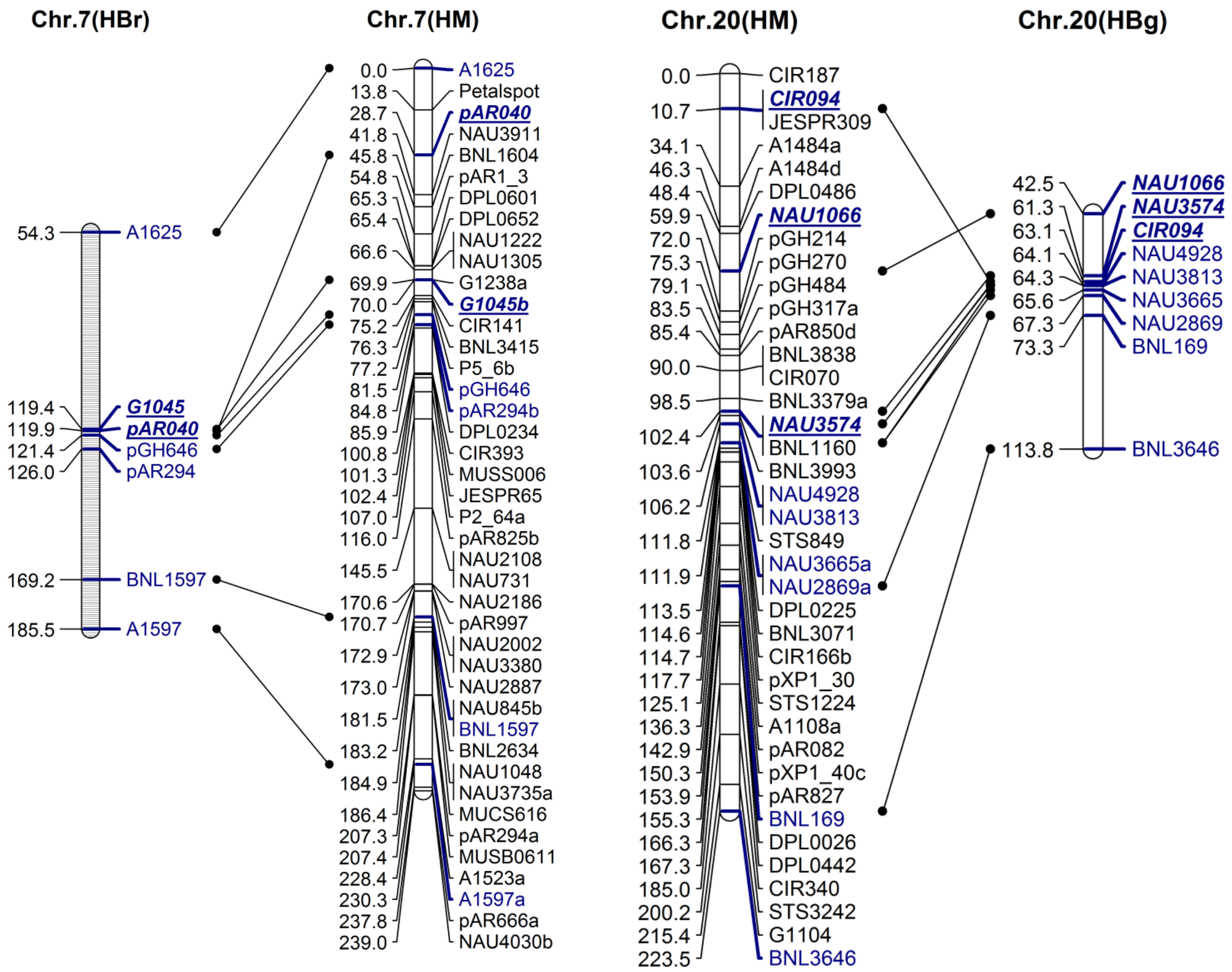

Chr.7(HBr)
Chr.7(HM)
Chr.20(HM)
Chr.20(HBg)
54.3 A1625
119.4 G1045
119.9 pAR040
121.4 pGH646
126.0 pAR294
169.2 BNL1597
185.5 A1597
0.0 A1625
13.8 Petalspot
28.7 pAR040
41.8 NAU3911
45.8 BNL1604
54.8 pAR1_3
65.3 DPL0601
65.4 DPL0652
66.6 NAU1222
NAU1305
69.9 G1238a
70.0 G1045b
75.2 CIR141
76.3 BNL3415
77.2 P5_6b
81.5 pGH646
84.8 pAR294b
85.9 DPL0234
100.8 CIR393
101.3 MUSS006
102.4 JESPR65
107.0 P2_64a
116.0 pAR825b
145.5 NAU2108
NAU731
170.6 NAU2186
170.7 pAR997
172.9 NAU2002
NAU3380
173.0 NAU2887
NAU845b
181.5 BNL1597
183.2 BNL2634
184.9 NAU1048
NAU3735a
186.4 MUCS616
207.3 pAR294a
207.4 MUSB0611
228.4 A1523a
230.3 A1597a
237.8 pAR666a
239.0 NAU4030b
0.0 CIR187
10.7 CIR094
JESPR309
34.1 A1484a
46.3 A1484d
48.4 DPL0486
59.9 NAU1066
72.0 pGH214
75.3 pGH270
79.1 pGH484
83.5 pGH317a
85.4 pAR850d
90.0 BNL3838
CIR070
98.5 BNL3379a
102.4 NAU3574
BNL1160
103.6 BNL3993
NAU4928
106.2 NAU3813
111.8 STS849
NAU3665a
111.9 NAU2869a
113.5 DPL0225
114.6 BNL3071
114.7 CIR166b
117.7 pXP1_30
125.1 STS1224
136.3 A1108a
142.9 pAR082
150.3 pXP1_40c
153.9 pAR827
155.3 BNL169
166.3 DPL0026
167.3 DPL0442
185.0 CIR340
200.2 STS3242
215.4 G1104
223.5 BNL3646
42.5 NAU1066
61.3 NAU3574
63.1 CIR094
64.1 NAU4928
64.3 NAU3813
65.6 NAU3665
67.3 NAU2869
73.3 BNL169
113.8 BNL3646

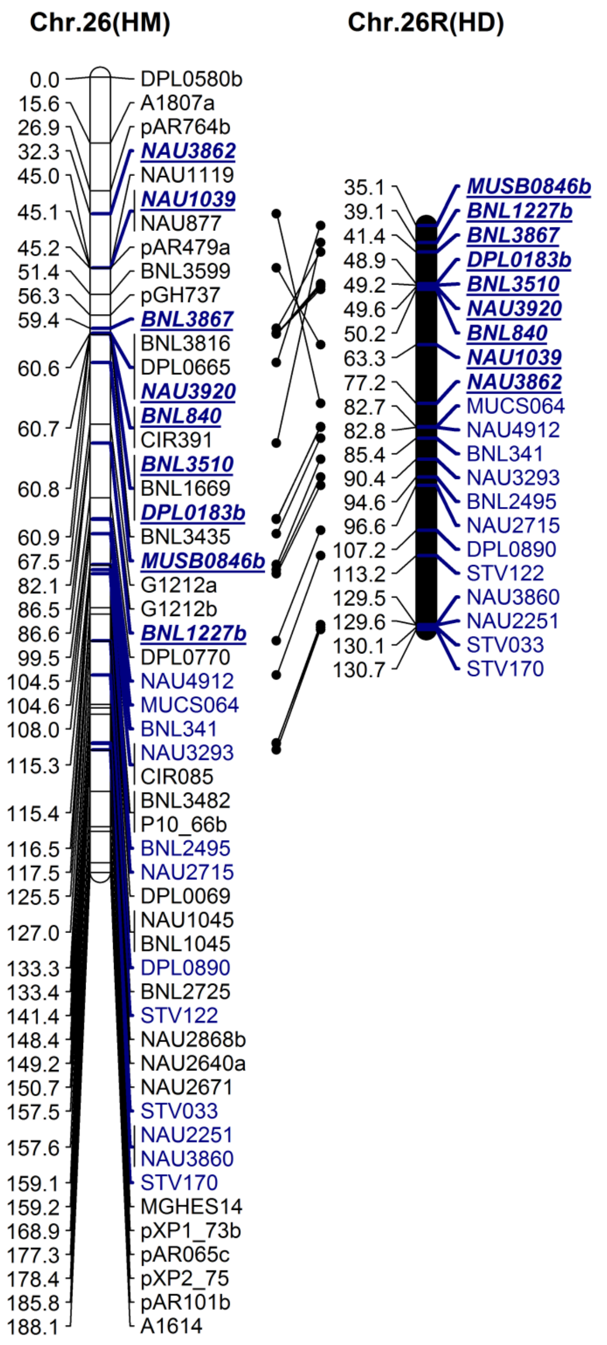

Figure S1 Comparison between *Gossypium hirsutum* × *G. mustelinum* (HM) genetic map with maps of *G. hirsutum* × *G. tomentosum* (HT), *G. hirsutum* × *G. barbadense* (HB), and *G. hirsutum* × *G. darwinii* (HD). The “R” following chromosome name means that this chromosome was drawn upside down so as to make comparison easier to see. HBg means the HB map information from Guo et al. (2007), HBr means the HB map information from Rong et al. (2004). The maps are drawn in Kosambi centiMorgans. Anchor loci segregated in the HM and HT maps are colored and connected by solid lines, and the anchor loci involved in the inversions are underlined, bold and italic. Only the chromosomes detected inversions between HM and other maps are shown in the figure.
